# Fluorescent Supramolecular Polymers Formed by Crown Ether-Based Host-Guest Interaction

**DOI:** 10.3389/fchem.2020.00560

**Published:** 2020-07-24

**Authors:** Jinjin Zhang, Huayu Qiu, Tian He, Yang Li, Shouchun Yin

**Affiliations:** ^1^College of Material, Chemistry and Chemical Engineering, Hangzhou Normal University, Hangzhou, China; ^2^Key Laboratory of Organosilicon Chemistry and Materials Technology of Ministry of Education, Hangzhou Normal University, Hangzhou, China

**Keywords:** supramolecular polymer, fluorescence, crown ether, host-guest interaction, supramolecular coordination complex

## Abstract

Inspired by the vast array of assemblies present in nature, supramolecular chemistry has attracted significant attention on account of its diverse supra-structures, which include micelles, vesicles, and fibers, in addition to its extensive applications in luminescent materials, sensors, bioimaging, and drug delivery over the past decades. Supramolecular polymers, which represent a combination of supramolecular chemistry and polymer science, are constructed by non-covalent interactions, such as host-guest interactions, hydrogen bonding, hydrophobic or hydrophilic interactions, metal-ligand interactions, π-π stacking, and electrostatic interactions. To date, numerous host-guest recognition systems have been reported, including crown ethers, cyclodextrins, calixarenes, cucurbituril, pillararenes, and other macrocyclic hosts. Among them, crown ethers, as the first generation of macrocyclic hosts, provide a promising and facile alternative route to supramolecular polymers. In addition, the incorporation of fluorophores into supramolecular polymers could endow them with multiple properties and functions, thereby presenting potential advantages in the context of smart materials. Thus, this review focuses on the fabrication strategies, interesting properties, and potential applications of fluorescent supramolecular polymers based on crown ethers. Typical examples are presented and discussed in terms of three different types of building blocks, namely covalently bonded low-molecular-weight compounds, polymers modified by hosts or guests, and supramolecular coordination complexes.

**Graphical Abstract:**
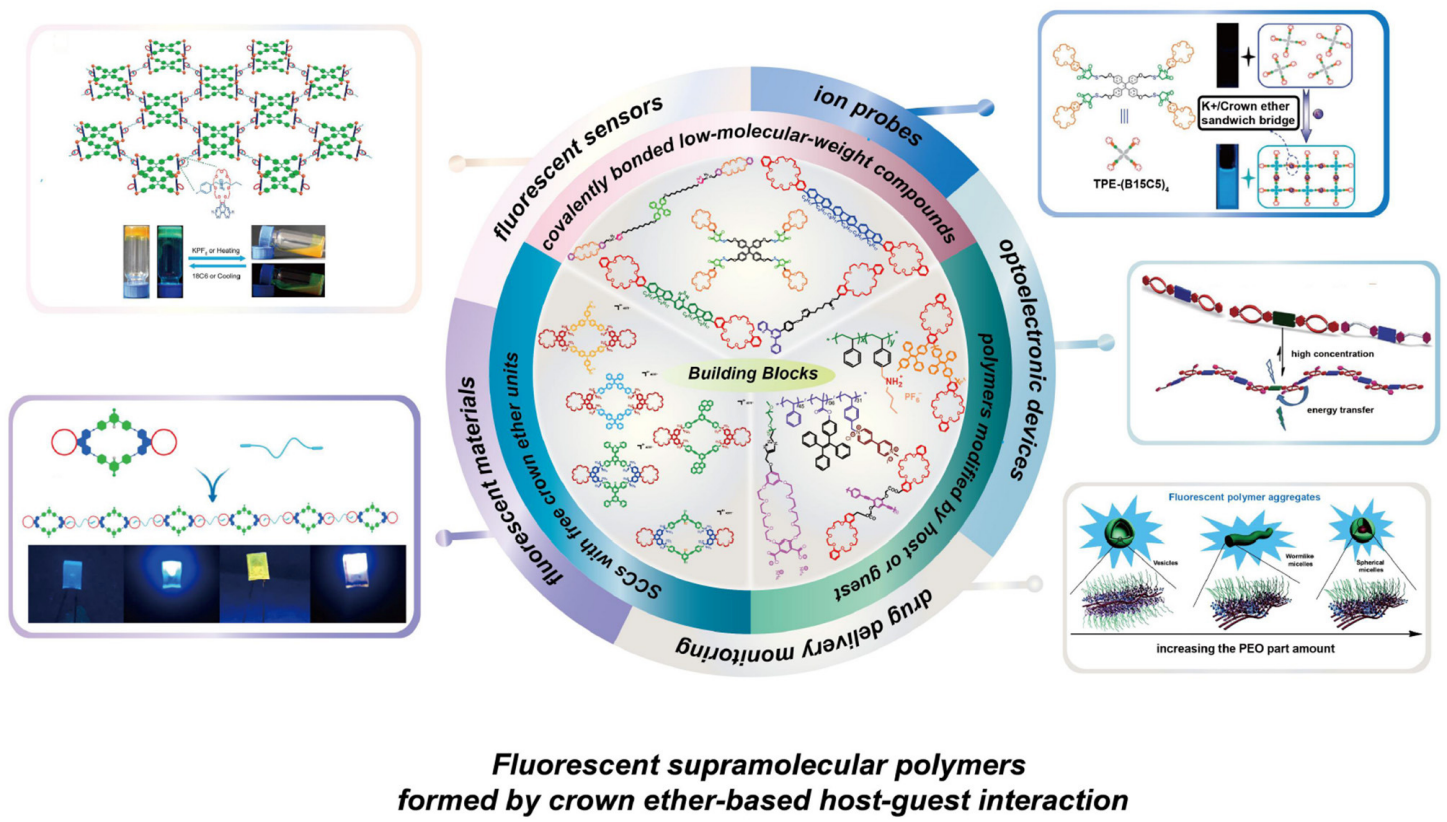
The building blocks and applications of fluorescent supramolecular polymers formed by crown ether-based host-guest interaction.

## Introduction

Molecular self-assembly is the key to obtaining complicated biomolecules in natural systems, such as proteins, nucleic acids, phospholipid membranes, ribosomes, and microtubules (Chen et al., 2016; Laurent et al., 2017; Sun et al., 2019). Drawing inspiration from the vast number of assemblies present in nature, functional materials for applications in luminescent materials, sensors, bioimaging, and drug delivery (Chen L. -J. et al., 2015; Yan et al., 2015; Zhang M. et al., 2016; Yu et al., 2017; Zhang et al., 2017a) have been obtained through molecular self-assembly to form diverse supra-structures, such as micelles, vesicles, ribbons, and fibers (Ji et al., 2013a; Yan et al., 2013; Shi et al., 2016). In contrast to molecules based on traditional covalent bonds, supramolecular self-assembled structures exhibit specific characteristics, such as self-healing, coordinability, and responsiveness to stimuli, due to the dynamic and reversible nature of the non-covalent bonds or dynamic covalent bonds (Yan et al., 2012a; Yu et al., 2013, 2014; Zhang et al., 2018, 2019a; Deng et al., 2020). Among them, supramolecular polymers, in which repeating units are held together to form polymeric arrays through intermolecular bonds (e.g., host-guest interactions, hydrogen bonding, hydrophobic/hydrophilic interactions, metal-ligand interactions, and π-π stacking) are considered to be promising smart materials.

To date, host-guest recognition systems have been widely employed to construct supramolecular polymers, where the hosts are often crown ethers, cyclodextrins, calixarenes, cucurbituril, pillararenes, and other macrocyclic hosts (Ma and Zhao, 2015; Qu et al., 2015; Yu et al., 2015; Liu et al., 2018; Shi et al., 2019). Among them, crown ethers, which were the first artificial macrocycles, are a type of macrocyclic polyether containing multiple oxygen methylene units, with examples including 18-crown-6, 21-crown-7, 24-crown-8, and other analogous derivatives, which can be host to positive ions and neutral molecules (Yamaguchi et al., 1998; Gibson et al., 2002; Huang and Gibson, 2004; Wei et al., 2015). In 1967, Pedersen reported, for the first time, stable complexes formed by crown ethers and certain cations that interacted via ion-dipole interactions between the cations and the high electron-density oxygen atoms of the crown ethers (Pedersen, 1967). Other studies have focused on the recognition between crown ethers and metal cations, such as K^+^, Li^+^, and Na^+^ (Pedersen, 1967; Ma et al., 2015); however, in the wake of in-depth studies, complexes constructed from crown ethers and organic cations or organic neutral molecules were discovered, with examples including secondary ammonium salts, diazonium salts, and paraquat (Yamaguchi and Gibson, 1999; Gibson et al., 2003; Huang et al., 2007; Zhang et al., 2007). Crown ethers are known to accommodate a variety of guests, and they tend to exhibit strong binding affinities for specific guests due to their multidentate structure and relatively strong non-covalent interactions. Therefore, host-guest recognition systems based on crown ethers offer distinct advantages in terms of fabricating supramolecular polymers (Yan et al., 2012b; Zheng et al., 2012; Ding et al., 2013; Li X. et al., 2018; Li et al., 2019b; Xiao et al., 2020). Importantly, the formation of supramolecular polymers can overcome issues related to the preparation of traditional polymers, since the latter methods tend to require an auxiliary initiator, high temperatures, and long reaction times. Furthermore, the introduction of host-guest interactions can also endow the constructed supramolecular polymers with dynamic and reversible properties (Dong et al., 2012; Yan et al., 2014; Zhan et al., 2014a,b; Huang et al., 2018; Wang et al., 2020). For example, Dong and co-workers made a supramolecular polymer network with dynamic reversibility, good malleability, and processability that depended on the formation of host-guest interactions between crown ethers and ammonium motifs of H2G2-type monomer (Wang et al., 2020). Moreover, recently, a crown ether-based interaction has been used to enhance the mechanical strength of a supramolecualr polymer, which is promising for more exciting applications (Shi et al., 2020). Although a range of supramolecular polymers have been constructed based on host-guest interactions, the development of crown ether-based supramolecular polymers with additional multiple functionalities remains of interest.

Fluorescence refers to a cold luminescence phenomenon cause by photoluminescence, which is light emitted by a substance after it absorbs light or other electromagnetic radiation. In recent years, fluorescent materials have been widely used in life and material science. Fluorophores, which can emit fluorescence, are often incorporated into supramolecular polymers, since the resulting polymers inherit the fluorescence properties of the fluorogens in addition to exhibiting the dynamic and reversible properties originating from the non-covalent interactions. This renders them capable of exhibiting a fluorescence response to various external stimuli (Dong et al., 2016; Li et al., 2016, 2020; Wang et al., 2017). Fluorescent supramolecular polymers have therefore been widely applied in fluorescent materials, fluorescent probing, data storage, bio-imaging, drug delivery, and cancer therapy (Lou and Yang, 2018; Li et al., 2019a). Indeed, various kinds of organic fluorogens exist, such as coumarins, fluoresceins, cyanine naphthalimide rhodamine, conjugated polymer groups, and aggregation-induced emission (AIE) luminogens (Dsouza et al., 2011; Fermi et al., 2014; Ma et al., 2016; Peng et al., 2017; Li Y. et al., 2018). In addition, when conventional organic chromophores were combined with supramolecular polymers, the resulting polymers were found to exhibit weak fluorescence, since the formation of supramolecular polymers must be carried out at high concentrations, thereby causing aggregation-caused quenching (ACQ) of the traditional fluorescent chromophore. This issue can be efficiently resolved through the use of AIE luminogens, which were initially developed by Luo et al. (2001) and were found to exhibit faint luminescence in a dilute solution but strong luminescence in the solid or aggregate state due to the restriction of intramolecular vibrations and rotations. Furthermore, Lou and Yang focused on the combination of AIEgens with supramolecular macrocyclics, with a recent review of the supra-structures obtained, which included supramolecular nanocomplexes/polymers, supramolecular nanoparticles, and host-guest complexes on nanosurfaces (Lou and Yang, 2018). Thus, in the next parts of this review, we will focus on fluorescent supramolecular polymers based on crown ethers, which are not confined to AIE-active luminogens, and discuss their fabrication strategies, interesting properties, and potential applications. In addition, we present diverse methods for combining free crown ether units with various building blocks, including covalently bonded low-molecular-weight compounds, polymers modified by hosts or guests, and supramolecular coordination complexes (SCCs), and representative examples are scrutinized over a comprehensive scope.

## Crown Ether-Based Fluorescent Supramolecular Polymers Constructed by Covalently Bonded Low-Molecular-Weight Compounds

The most efficient and straightforward method of constructing fluorescent supramolecular polymers is to design and prepare fluorescent hosts or guests through the rational chemical modification of chromophores. Subsequently, certain crown ethers and guests are brought together through host-guest interactions along with π-π stacking (Ma et al., 2015) and/or donor-acceptor interactions (Roy et al., 2016).

**Figure 1:**
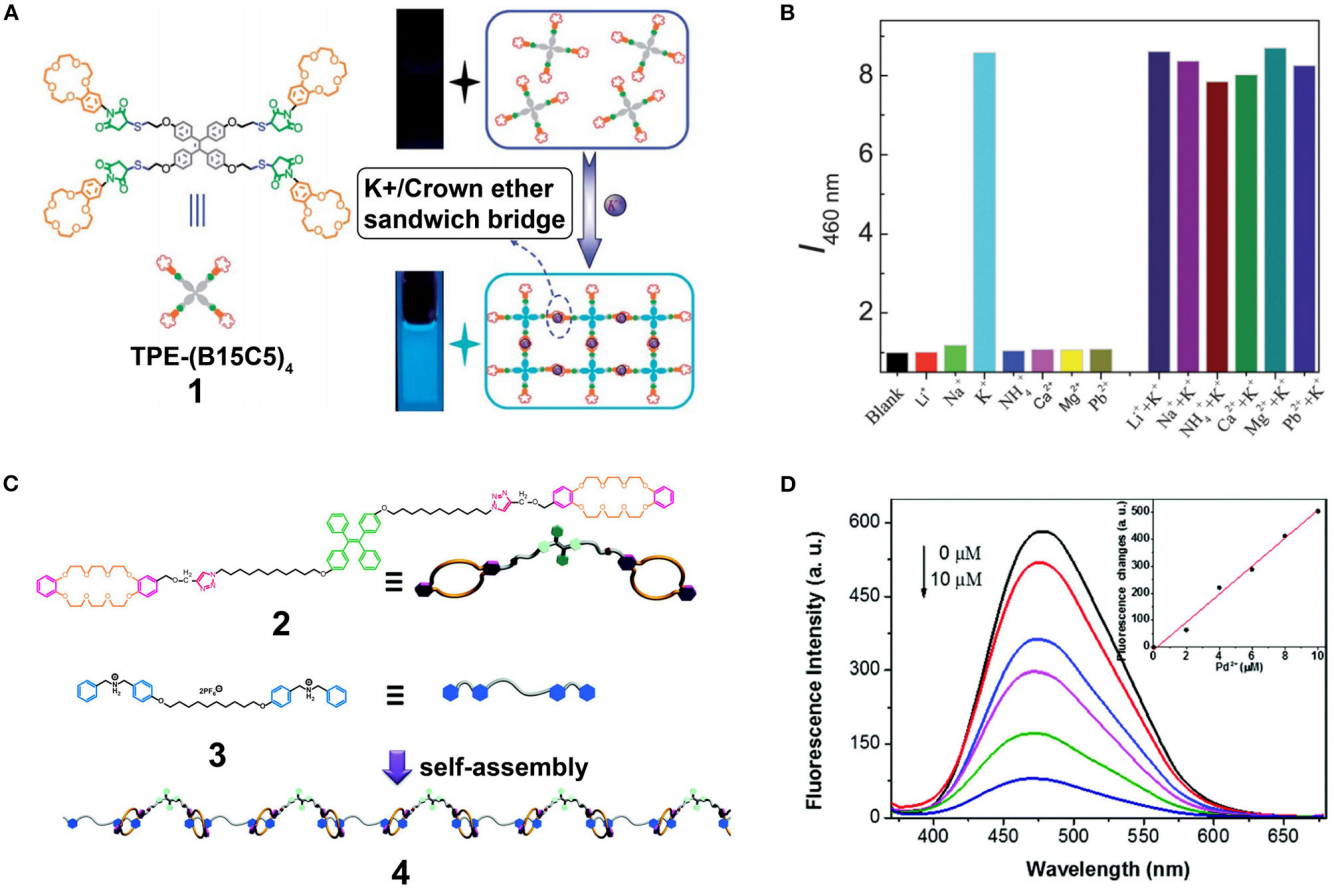
**(A)** Schematic illustration of the AIE of TPE-(B15C5)_4_ induced by recognition between the crown ether moieties and K^+^ ions. **(B)** Relative fluorescence intensity at λ_em_ = 460 nm of TPE-(B15C5)_4_ in THF solution recorded under the addition of K^+^ ions in the absence or presence of other interfering ions (i.e., Li^+^, Na^+^, ${\text{NH}}_{4}^{+}$, Ca^2+^, Mg^2+^, and Pb^2+^) (λ_ex_ = 360 nm; slit width: Ex. 5 nm, Em. 5 nm; 25°C). Adapted with permission from Wang et al. (2012); copyright 2012, Royal Society of Chemistry. **(C)** Cartoon representations of the formation of the linear fluorescent supramolecular polymer **4** from host **2** and guest **3**. **(D)** Fluorescence emission spectra of **4** with different concentrations of Pd^2+^ (0–10 μM) in the solid state (λ_ex_ = 350 nm). Inset: Linear relationship between the fluorescence intensity of **4** and the concentration of Pd^2+^ at λ_em_ = 577 nm. Adapted with permission from Chen D. et al. (2015); copyright 2015, Royal Society of Chemistry.

More specifically, Wang et al. reported the application of AIE-active supramolecular polymers based on the recognition system of crown ethers (Wang et al., 2012). They designed a novel and effective fluorometric K^+^ probe via host-guest molecular recognition and aggregation-induced emission. The host molecule TPE-(B15C5)_4_ (**1**) was synthesized by the functionalization of a four peripheral benzo-15-crown-5 (B15C5) with tetraphenylethylene (TPE) as the core (Figure 1A). The AIE feature of the TPE cores and the formation of cross-linked supramolecular polymers resulted in the aggregation of **1** and an enhancement in fluorescence emission; this system was suitable for application in K^+^ detection. Moreover, **1** exhibited an excellent selectivity toward K^+^ compared to other interfering ions (e.g., Li^+^, Na^+^, ${\text{NH}}_{4}^{+}$, Ca^+^, Mg^+^, and Pd^2+^) (Figure 1B), with a detection limit of ~1.0 μM. This work opened a new avenue for the development of sensitive and selective fluorometric off-on probes. Similarly, Chen D. et al. (2015) designed monomer **2**, wherein the host TPE core was linked with two dibenzo-24-crown-8 (DB24C8) and two 1,2,3-trizole units, and a dibenzylammonium (DBA) salt monomer **3** was employed as the guest. Consequently, an AIE-active supramolecular polymer **4** (Figure 1C) was obtained through host-guest interactions by mixing monomers **2** and **3** in a 1:1 molar ratio. The characteristic AIE properties of supramolecular polymer **4** originated from restriction of the TPE group intramolecular rotational motion. These properties were confirmed by observation that the fluorescence intensity of **4** was 6-fold stronger than that of monomer **1** at the same concentration. In addition, the enhanced fluorescence intensity of **4** was accompanied by a 13-nm red-shift upon increasing its concentration from 75 to 200 mM. Furthermore, the fluorescence intensity of **4** in the solid state was further increased along with a 79-nm blue-shift due to morphological changes in the aggregates. Interestingly, the fluorescence intensity of **4** decreased linearly upon the addition of Pd^2+^ (Figure 1D) owing to coordination interactions and energy transfer between the 1,2,3-trizole units and Pd^2+^. Thus, **4** can be employed as a Pd^2+^ fluorescent probe in the solid state, thereby enriching the application of supramolecular polymeric materials.

**Figure 2:**
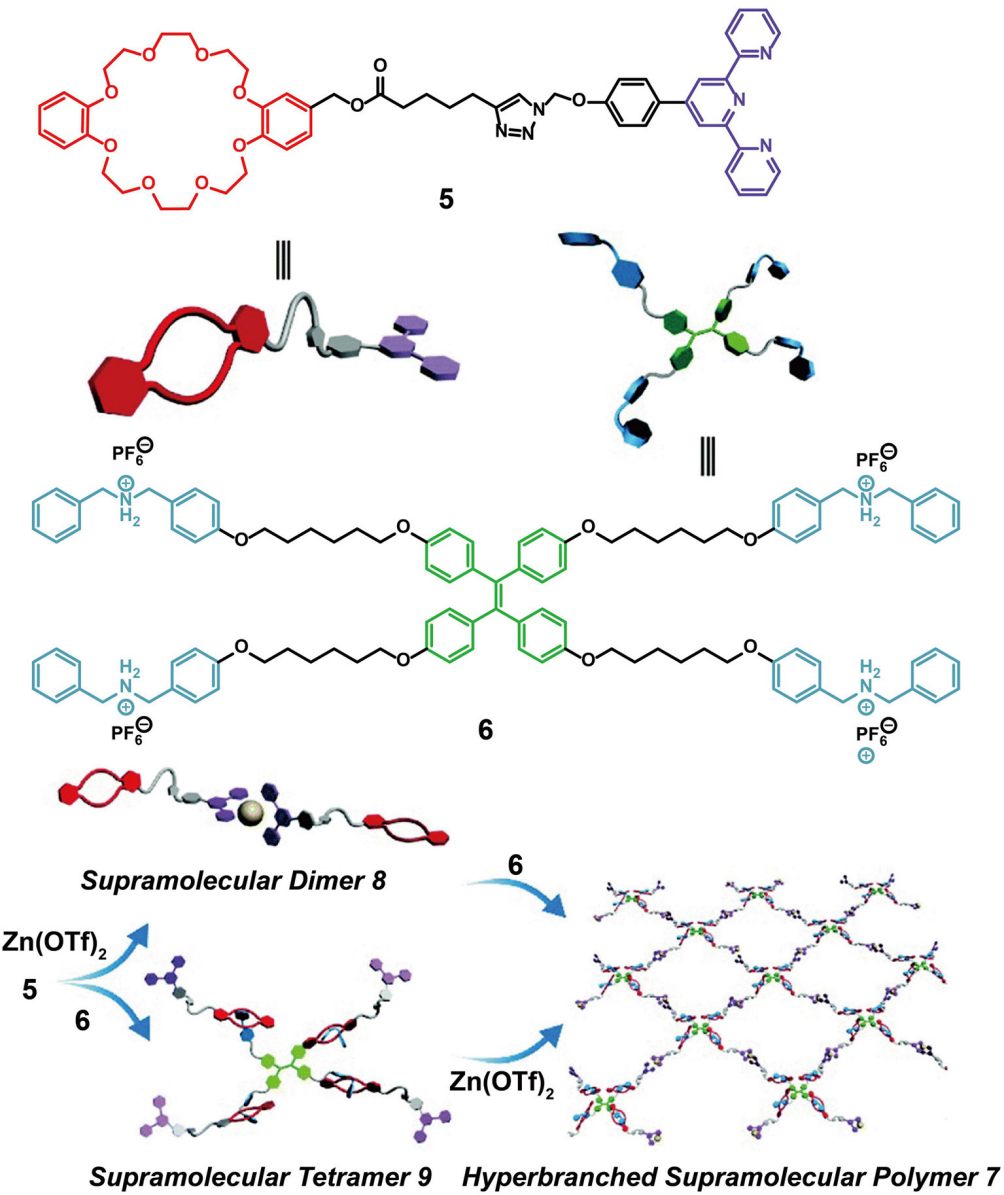
Schematic illustration of monomers **5** and **6** and cartoon representations of the formation of supramolecular dimer **8**, supramolecular tetramer **9**, and hyperbranched supramolecular polymer **7**. Adapted with permission from Zhang J. et al. (2016); copyright 2016, Royal Society of Chemistry.

In addition to their application as ion sensors, crown ether-based fluorescent supramolecular polymers formed using TPE-functionalized monomers can also be employed as smart and adaptive luminescent materials. For example, Zhang J. et al. (2016) designed two monomers, one (**5**) with a DB24C8 group at one end and a terpyridine moiety at the other end, and another (**6**) with a TPE core and four outer DBA salts. A hyperbranched fluorescent supramolecular polymer, **7**, was constructed through the connection of Zn(OTf)_2_ with monomers **5** and **6** via terpyridine-based metal-ligand interactions and crown ether-based host-guest interactions (Figure 2). The hyperbranched fluorescent supramolecular polymer **7** displayed a strong emission, while supramolecular dimer **8** and supramolecular tetramer **9**, which were formed by only one kind of non-covalent interaction, exhibited a reduced luminescence due to inefficient restriction of the intramolecular rotation/torsion of the TPE groups in **8** and **9**. In addition, **7** exhibited responsiveness to multiple stimuli, including temperature, pH, K^+^, and Cl^−^, due to the dynamic and reversible nature of the two orthogonal non-covalent interactions. Coincidentally, a further pH-responsive supramolecular polymer was fabricated by Bai et al. (2015) based on a similar principle, whereby the polymer consisted of TPE entities bearing DB24C8 or DBA on opposite phenyl rings. These results therefore provide a representative demonstration of self-assembly-induced emission (SAIE), which is conducive to the development of novel supramolecular materials exhibiting a stimulus-responsive fluorescence transition.

**Figure 3:**
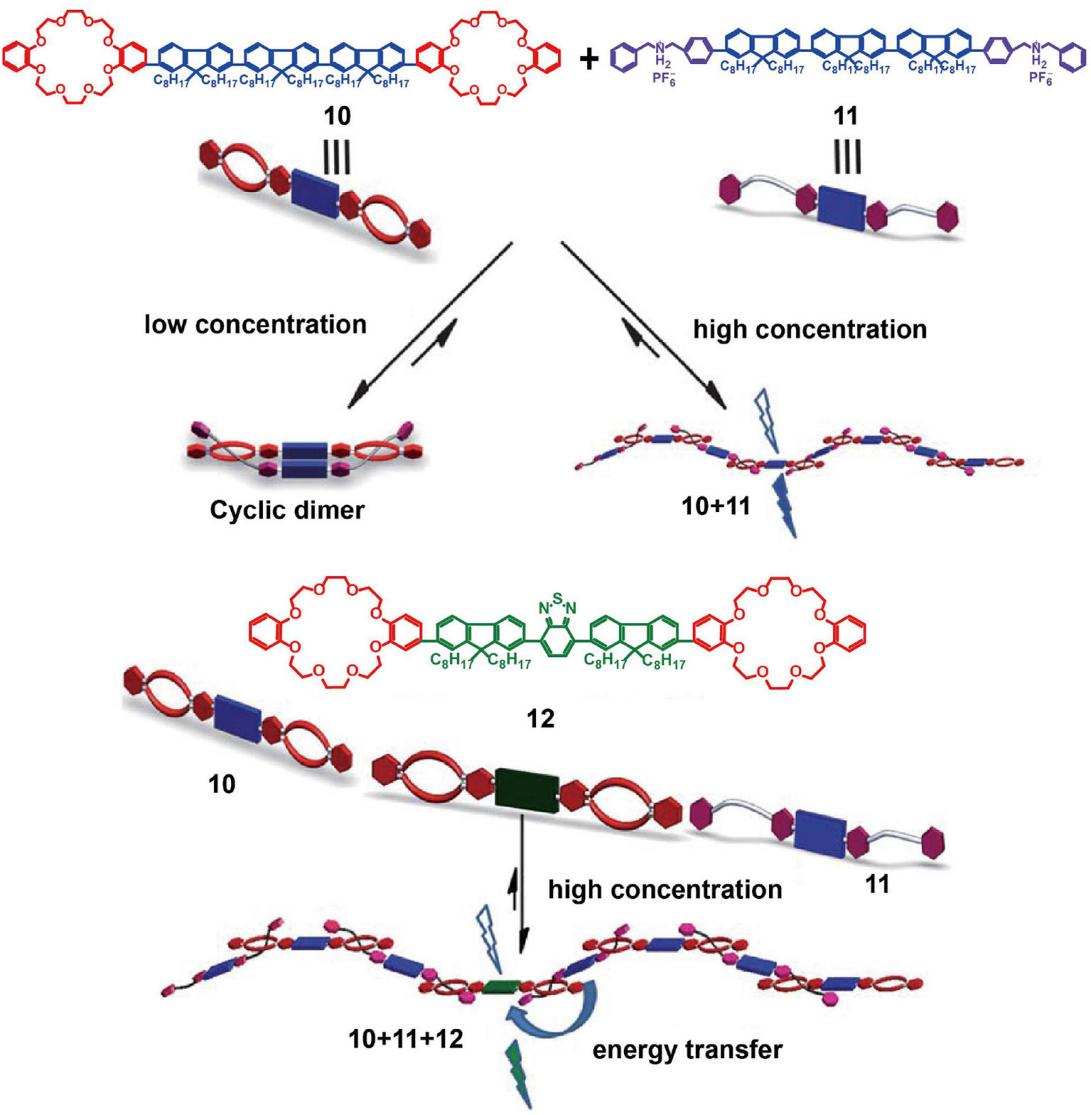
Cartoon representations of the formation of SLEPs from host **10**, guest **11**, and doping host **12**. Adapted with permission from Zhang et al. (2012); copyright 2012, Royal Society of Chemistry.

In addition to the introduction of AIEgens such as TPE as luminescent groups, the use of fluorescent conjugated oligomers would also present potential advantages in the development of optoelectronic devices or fluorescence materials. For example, Zhang et al. (2012) reported supramolecular light-emitting polymers (SLEPs) prepared from blue-emitting conjugated oligomer **10** and green-emitting conjugated oligomer **12** as the hosts and blue-emitting conjugated oligomer **11** as the guest (Figure 3). The resulting polymer, based on host-guest interactions, exhibited good film formation abilities, a stable film morphology, and facile solution processability. Furthermore, the polymer emission was tuneable by controlling the content of dopant host **12**. More specifically, by doping 10 or 30% of **12** into the supramolecular system to promote efficient energy transfer among the oligomers, the resulting SLEPs showed a large red-shift photoluminescent emission, significantly enhanced photoluminescent efficiencies, and achieved superior device performance. Due to these advantages, SLEPs have the potential to promote the development of solution-processed optoelectronic devices.

**Figure 4:**
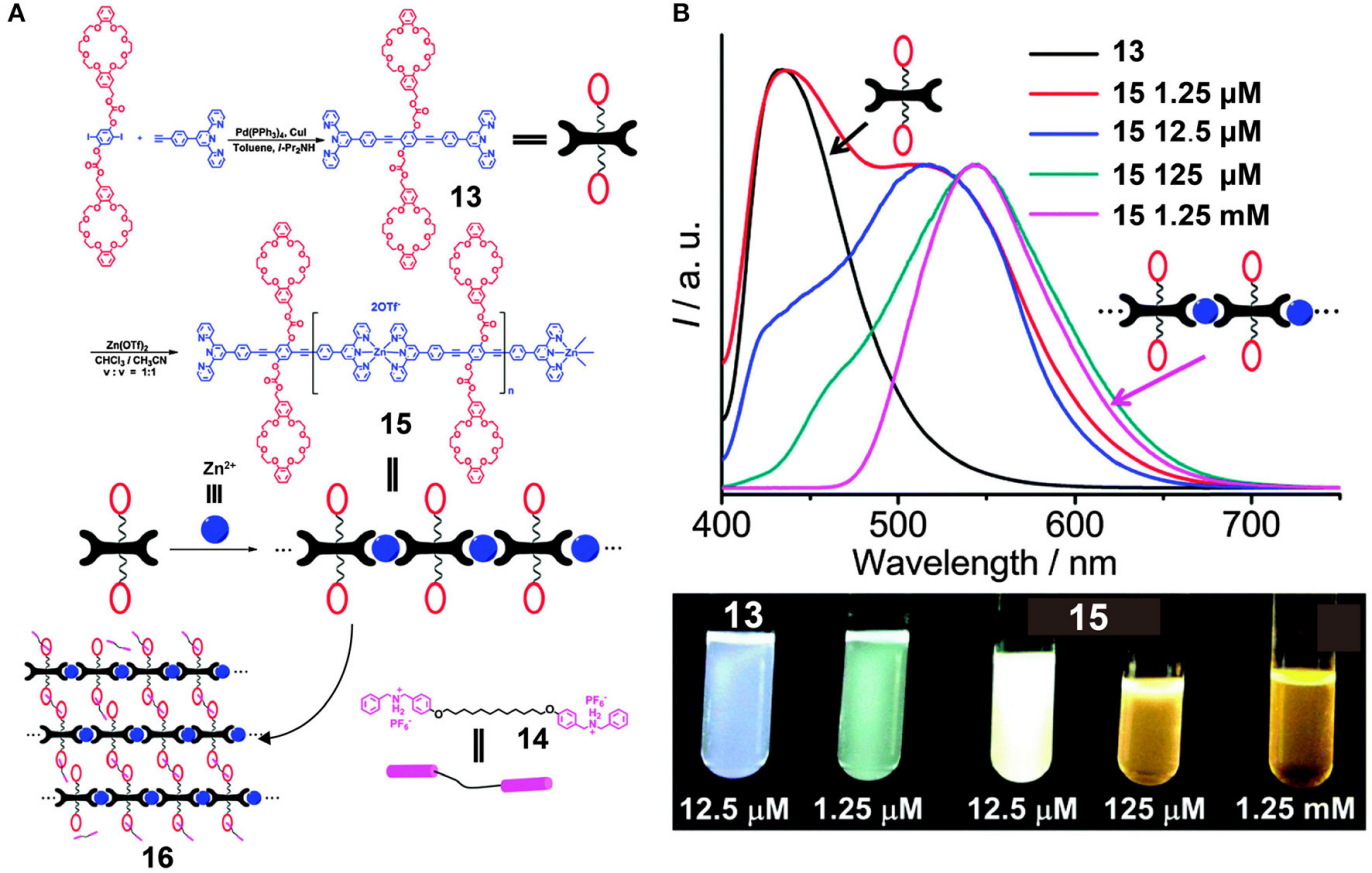
**(A)** Synthetic approaches and cartoon representations of the formation of **15** and **16**. **(B)** Fluorescence emission spectra of **13** and **15** in CHCl_3_/CH_3_CN (*v*/*v* = 1:1) and the corresponding optical photographs recorded under UV light (λ_ex_ = 365 nm). Adapted with permission from He et al. (2014); copyright 2015, Royal Society of Chemistry.

To endow supramolecular polymers with more versatile topological structures and increase their suitability for practical applications, He et al. (2014) constructed a cross-linked metallosupramolecular polymer, **16**, by employing metal-ligand interactions between conjugated bis-terpyridine ligand **13** and Zn^2+^ as the chromophore in addition to host-guest interactions between the two DB24C8 moieties in **13** and the two DBA groups in **14** (Figure 4A). The resulting linear conjugated supramolecular polymer **15** based on the terpyridine/Zn recognition motifs exhibited concentration-controllable emission varying in color from cyan to white to yellow (Figure 4B) due to the presence of components bearing different numbers of repeat units, including monomers, oligomers, and polymers. Interestingly, when the concentration of **15** is 12.5 μM, nearly white emission occurred in the absence of other complementary fluorescence groups. Moreover, upon increasing the quantities of added guest molecules **14** into **15**, the fluorescence intensity decreased gradually due to the formation of cross-linked metallosupramolecular polymer **16** via host-guest interactions between the DB24C8 moieties and the DBA moieties. In addition, the fluorescence intensity of **16** varied with changes in pH, which renders **16** suitable for application in acid-base stimulus-responsive materials. With such dual responsiveness, this supramolecular polymer represents an important advance in the design of fluorescent materials and molecular devices.

## Crown Ether-Based Fluorescent Supramolecular Polymers Constructed Using Polymers

The construction of crown ether-based fluorescent supramolecular polymers using covalently bonded low-molecular-weight compounds has a number of advantages, including simple synthetic routes, clear molecular structures, and ease of building supramolecular polymers with complicated topological structures. However, the stabilities and mechanical properties of the resulting supramolecular polymers tend to be poor, and so to overcome this issue, the incorporation of covalently bonded polymers into the supramolecular polymeric systems is an option. Indeed, with the development of supramolecular chemistry and polymer science, growing numbers of tailor-made covalent polymers have been employed as building blocks to construct supramolecular polymers through non-covalent interactions, and these polymers inherit the properties of both covalent and non-covalent bonding, including the associated mechanical properties, photophysical properties, reversibility, and stimuli-responsiveness. In such cases, polymers can play the roles of the host, the guest, or both.

### Polymers as Hosts and Micromolecules as Guests

**Figure 5:**
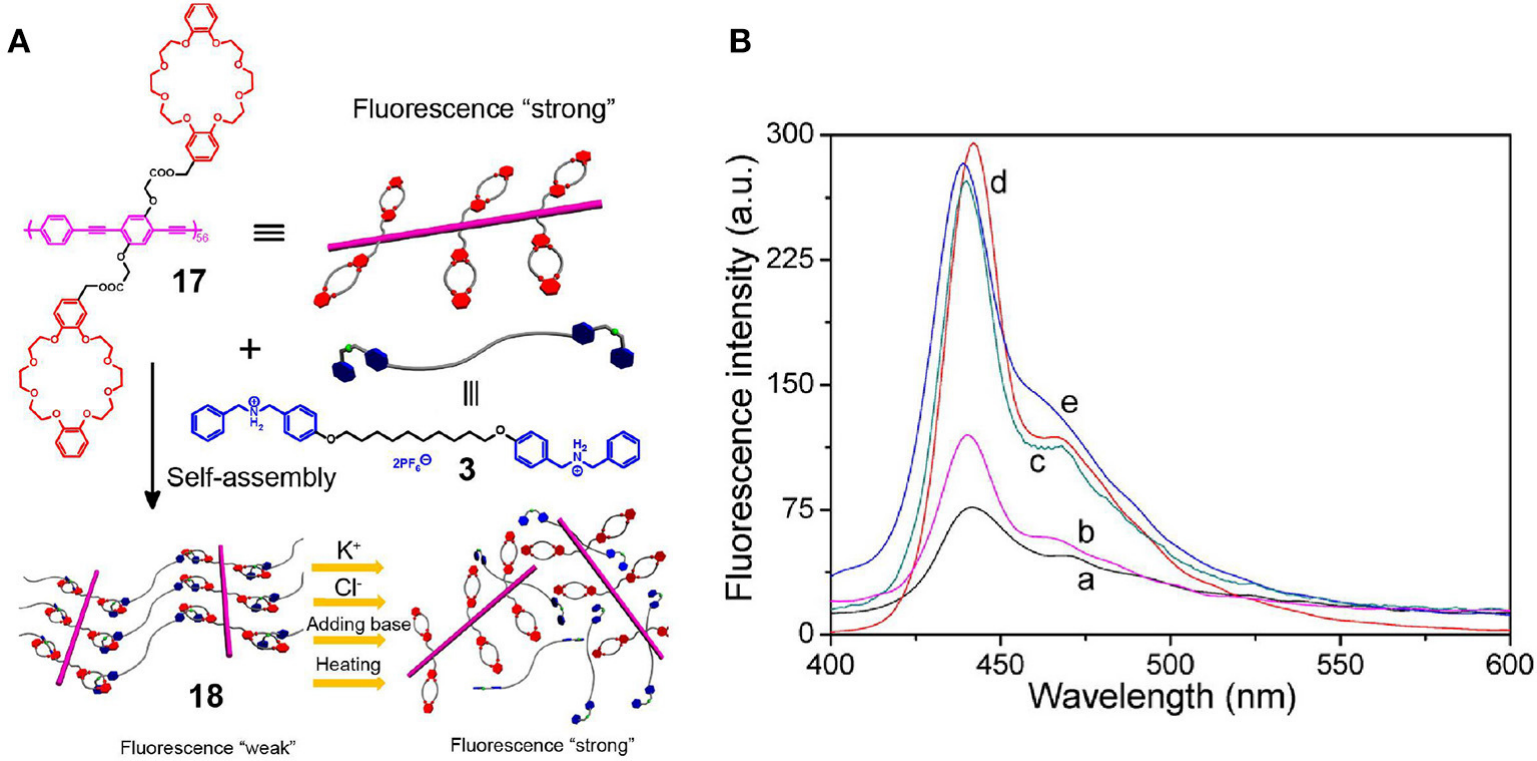
**(A)** Cartoon representations of the formation of the supramolecular cross-linked network **18** from polymer **17** and cross-linker **3**. **(B)** Fluorescence emission spectra of (a) **18** constructed using **17** (2.0 μM) and **3** (100 μM) and after (b) treatment by heating at 50°C and treatment with (c) 200 μM KPF_6_, (d) 200 μM TBACl, and (e) 200 μM Et_3_N. Adapted with permission from Ji et al. (2013b); copyright 2013, American Chemical Society.

In the case where polymers are employed as hosts and micromolecules as guests, crown ethers are modified into the traditional polymer hosts, while low-molecular-weight secondary ammonium salts, diazonium, salts, and paraquat are used as the guests. More specifically, Ji et al. (2013b) developed a supramolecular cross-linked network **18** via host-guest interactions through the use of fluorescent conjugated polymer chains grafted with the DB24C8 groups of **17** as the host and a bisammonium salt as the guest (**3**) (Figure 5A). Compared with the fluorescence intensity of polymer **17**, that of the supramolecular cross-linked network **18** was significantly lower due to the ACQ properties of the conjugated poly(phenylene ethynylene) polymeric backbones. The structure of polymer network **18** could be destroyed by multiple stimuli, such as variations in the temperature or pH change or the addition of K^+^ or Cl^−^ ions, and this was accompanied by an increase in the fluorescence intensity due to the presence of dynamic non-covalent bonds (Figure 5B). Interestingly, the cross-linked polymer network thin film emitted a stronger fluorescence upon exposure to an alkaline gas, such as ammonia vapor, since the deprotonation of cross-linker **3** resulted in dissociation of the aggregated state of the poly(phenylene ethynylene) polymer main chains. Hence, this system could be employed to probe various stimuli, and in particular, the presence of an alkaline gas, and so can be considered an attractive candidate for advanced sensor materials.

**Figure 6:**
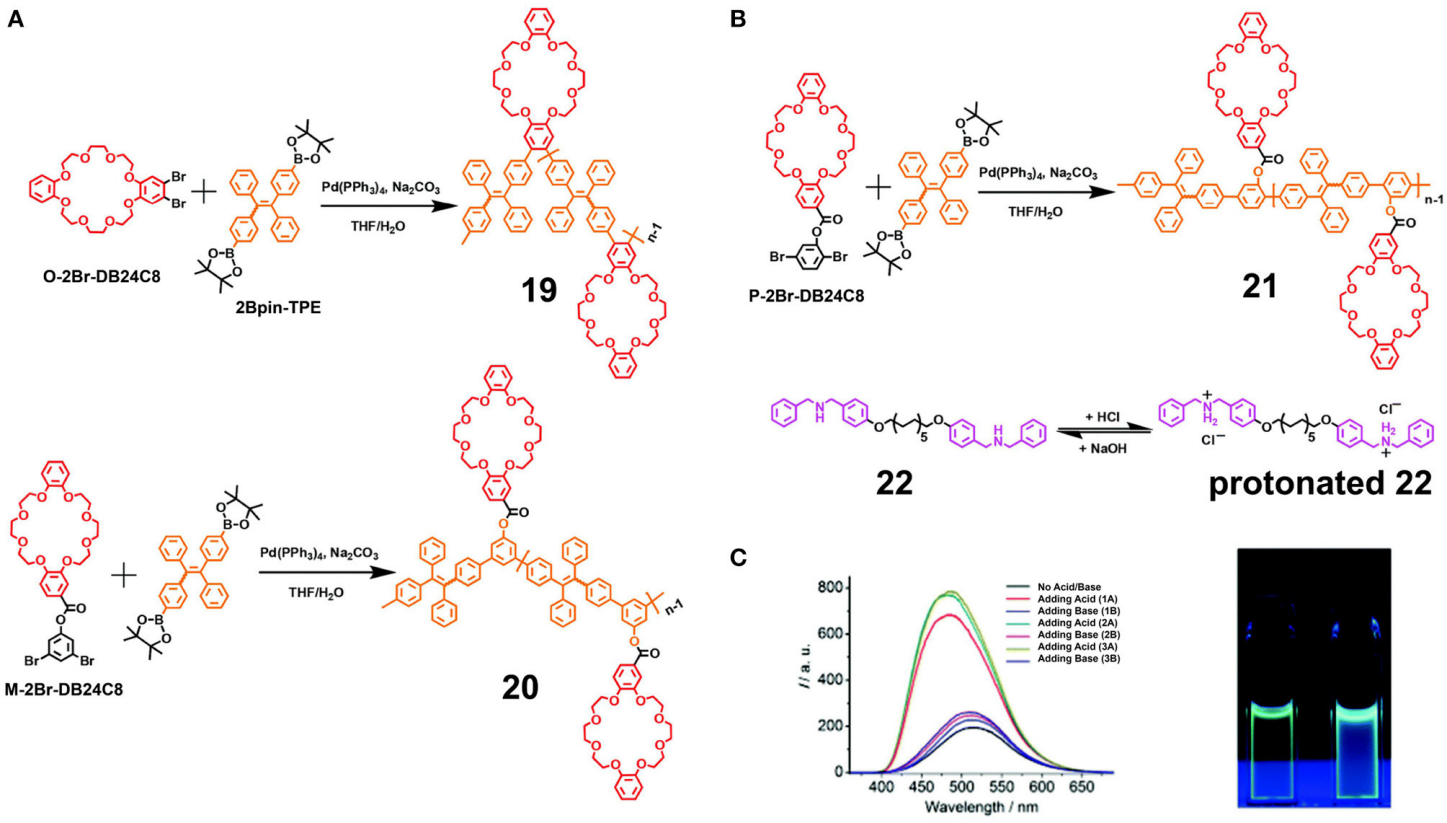
**(A)** Synthetic approaches to **19** and **20**. **(B)** Synthetic approach to **21** and the transition between **22** and protonated **22**. **(C)** Fluorescence emission spectra of **21/22** following three acid-base cycles, and the optical photographs of **21/22** upon the first treatment with HCl and NaOH recorded under UV light (λ_ex_ = 365 nm). Adapted with permission from He et al. (2016); copyright 2016, Royal Society of Chemistry.

To overcome the weak fluorescence exhibited by traditional conjugated polymers upon aggregation, He et al. (2016) constructed a series of crown ether-based fluorescent polymers **19–21** with AIE properties by coupling 2Bpin-TPE as an AIEgen with O-2Br-DB24C8, M-2Br-DB24C8, and P-2Br-DB24C8, in addition to a bisammonium salt **22** (Figures 6A,B). It should be noted here that the structural difference between polymers **19–21** was the linkage position of the TPE group. Thus, polymers **19–21** exhibited different degrees of aggregation-induced emission enhancement (AIEE) upon increasing the fraction of the poor solvent present in the mixture. For example, **19** exhibited a relatively bright emission in the dilute solution state, while its fluorescence enhancement was limited upon aggregation. This was accounted for by considering that the suppression of the intramolecular motion of the TPE moieties in **19** connected at the *ortho*-position was more efficient than the associated suppression in **20** at the *meta*-position and in **21** at the *para*-position. Interestingly, following treatment with three acid-base cycles, the system formed using **21** and **22** exhibited a highly reversible fluorescence intensity and emission wavelength (Figure 6C), while the systems formed from **19** and **22** or **20** and **22** exhibited stepwise increases in their fluorescence intensities. This was attributed to recognition between DB24C8 and DBAS and the efficient salting-out effect of NaCl generated from the acid-base process, which in turn facilitated morphological evolution from micelles to larger vesicles. However, due to the more rigid conformation of the polymer chain of **21**, the construction of vesicles from the **21/22** system was more challenging, and so stabilization of the fluorescence intensity was less efficient. Based on such findings, these polymers can be considered promising materials for use in the field of optoelectronic devices and fluorescent sensors.

### Polymers as Guests and Micromolecules as Hosts

**Figure 7:**
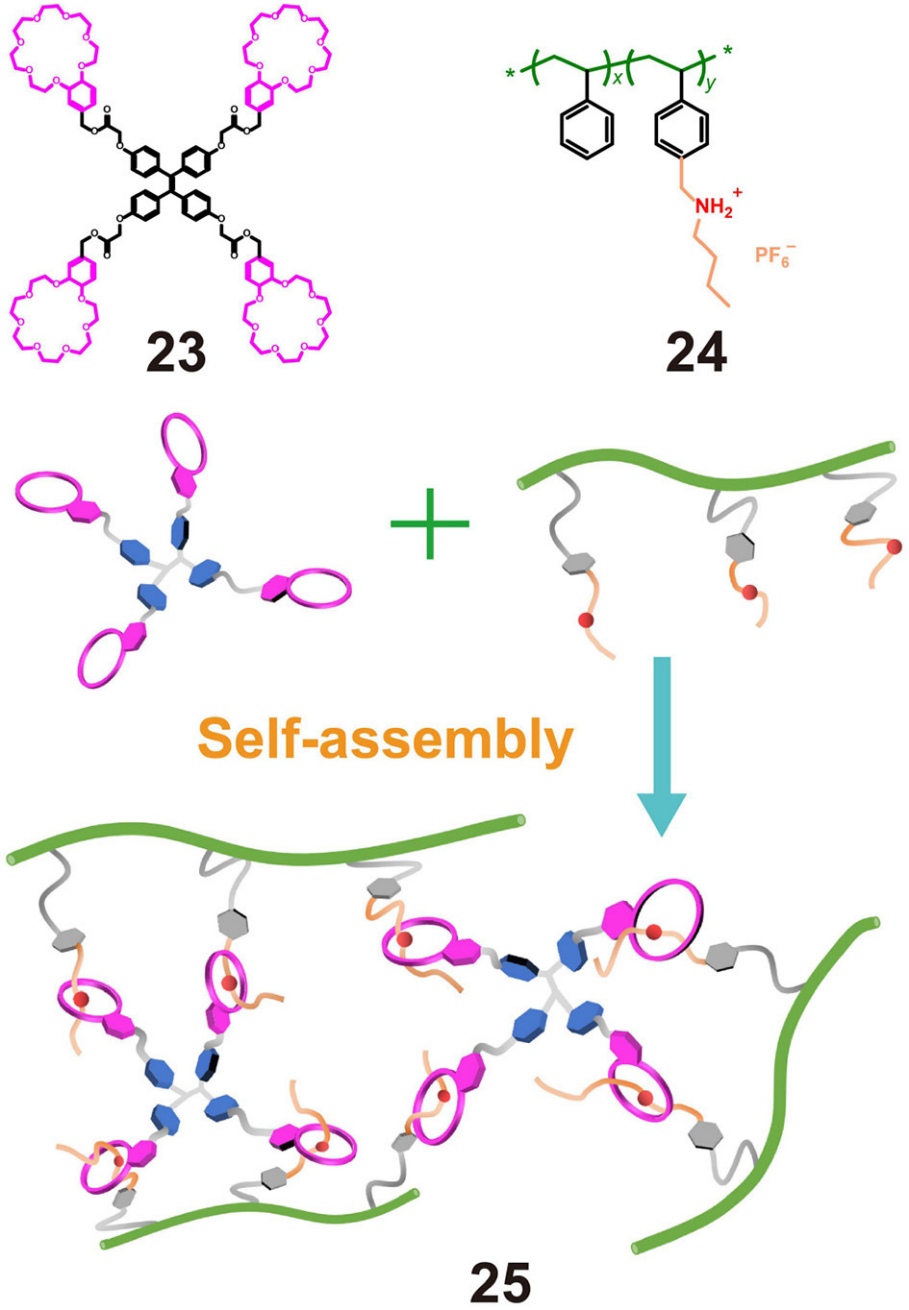
Chemical structures of **23** and **24** and a cartoon representation of the formation of fluorescent supramolecular cross-linked polymer gel **25**.

In the case were polymers are employed as guests and micromolecules as hosts, secondary ammonium salts are commonly hung on traditional polymers to make the guest, while low-molecular-weight crown ethers are employed as the hosts. For example, Ji X. F. et al. (2015) synthesized polystyrene polymer **24** bearing dialkylammonium salt moieties as pendent groups and benzo-21-crown-7 (B21C7) macrocycles **23** functionalized on the four arms of TPE molecules. Upon mixing **24** and **23**, a fluorescent supramolecular polymer gel was obtained via host-guest interactions between the B21C7 units and the dialkylammonium salts (Figure 7). The gel emitted a strong fluorescence, while a solution of **23** containing the same molar concentration of TPE units showed almost no fluorescence, thereby indicating that gelation induced the fluorescence emission. In addition, the presence of non-covalent interactions rendered the gel responsive to changes in temperature and pH. Thus, upon heating or with the addition of triethylamine, the recognition between B21C7 and the dialkylammonium salts was destroyed, resulting in disassembly of the gel and the formation of a sol, which was accompanied by fluorescence quenching. However, the recognition between B21C7 and the dialkylammonium salts was easily recovered by cooling or by the addition of trifluoroacetic acid. This work therefore provided a strategy for the construction of functional supramolecular polymers.

**Figure 8:**
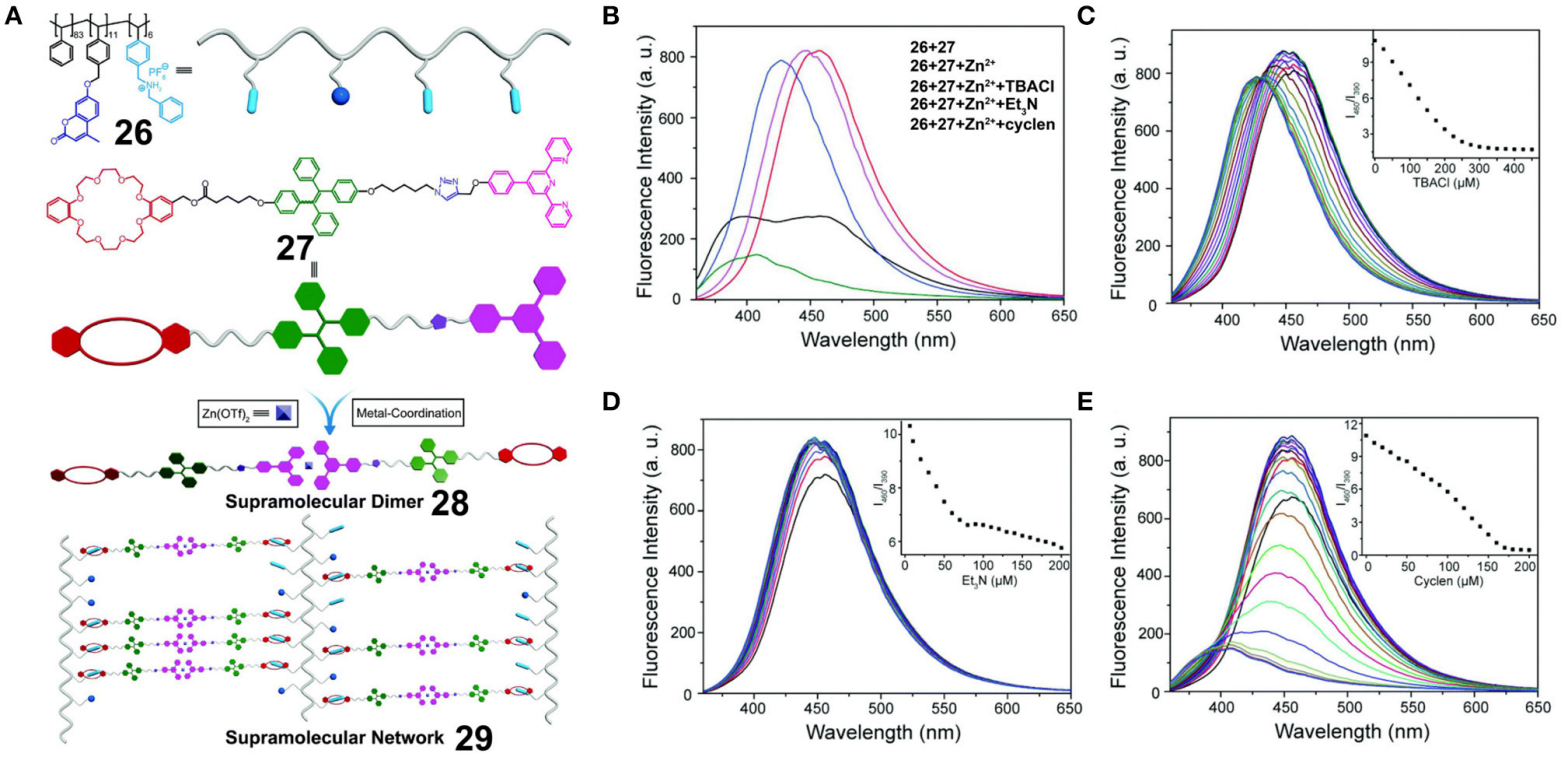
**(A)** Chemical structures of **26** and **27**, and cartoon representations of the formation of supramolecular dimer **28** and fluorescent supramolecular cross-linked polymer **29**. **(B)** Fluorescence emission spectra of the mixture of **26** (18.6 μM) and **27** (100 μM) and upon the addition of Zn(OTf)_2_ (50 μM), TBACl (450 μM), Et_3_N (200 μM), or cyclen (200 μM). Fluorescence emission spectra of the network upon the stepwise addition of **(C)** TBACl, **(D)** Et_3_N, and **(E)** cyclen. The insets show the plots of *I*_460_/*I*_390_ vs. the amount of **(C)** TBACl, **(D)** Et_3_N, and **(E)** cyclen added. Adapted with permission from Xu et al. (2018a); copyright 2018, Royal Society of Chemistry.

Employing the above-mentioned method, Xu et al. (2018a) prepared a polystyrene polymer **26** bearing coumarin moieties and dialkylammonium salts and combined this with a TPE derivative **27** bearing a DB24C8 unit and a terpyridine moiety at each side. A supramolecular network was constructed by the simple mixing of **26**, **27**, and Zn(OTf)_2_ through DB24C8/dialkylammonium salt and terpyridine/Zn recognition moieties (Figure 8A). Upon increasing the concentration of the system, the emission intensity of the supramolecular polymer network **29** at 460 nm (originating from the TPE units) was found to increase, while that at 390 nm (originating from the coumarin units) was found to decrease due to the AIE property of the TPE units and the ACQ property of the coumarin units. In this case, no obvious Förster resonance energy transfer (FRET) took place between **26** and **27** due to a lack of spectral overlap. Furthermore, the ratio between the emission intensities at 460 and 390 nm (*I*_460_/*I*_390_) varied linearly within a certain range upon the dissociation of **29** when NEt_3_, Cl^−^, or cyclen was added. Thus, the supramolecular polymer network **29** could be employed as a ratiometric sensor for pH, cyclen, and Cl^−^ with precise results (Figures 8B–E). Upon further increasing the concentration of the supramolecular polymer network **29**, a cross-linked supramolecular gel exhibiting multiple stimulus responsiveness and self-healing behavior was formed. Based on the above results, this fluorescent supramolecular polymer provided a representative illustration of a fluorescent material to serve as a ratiometric sensor, whereby monitoring was possible through self-calibration from two emission peaks.

**Figure 9:**
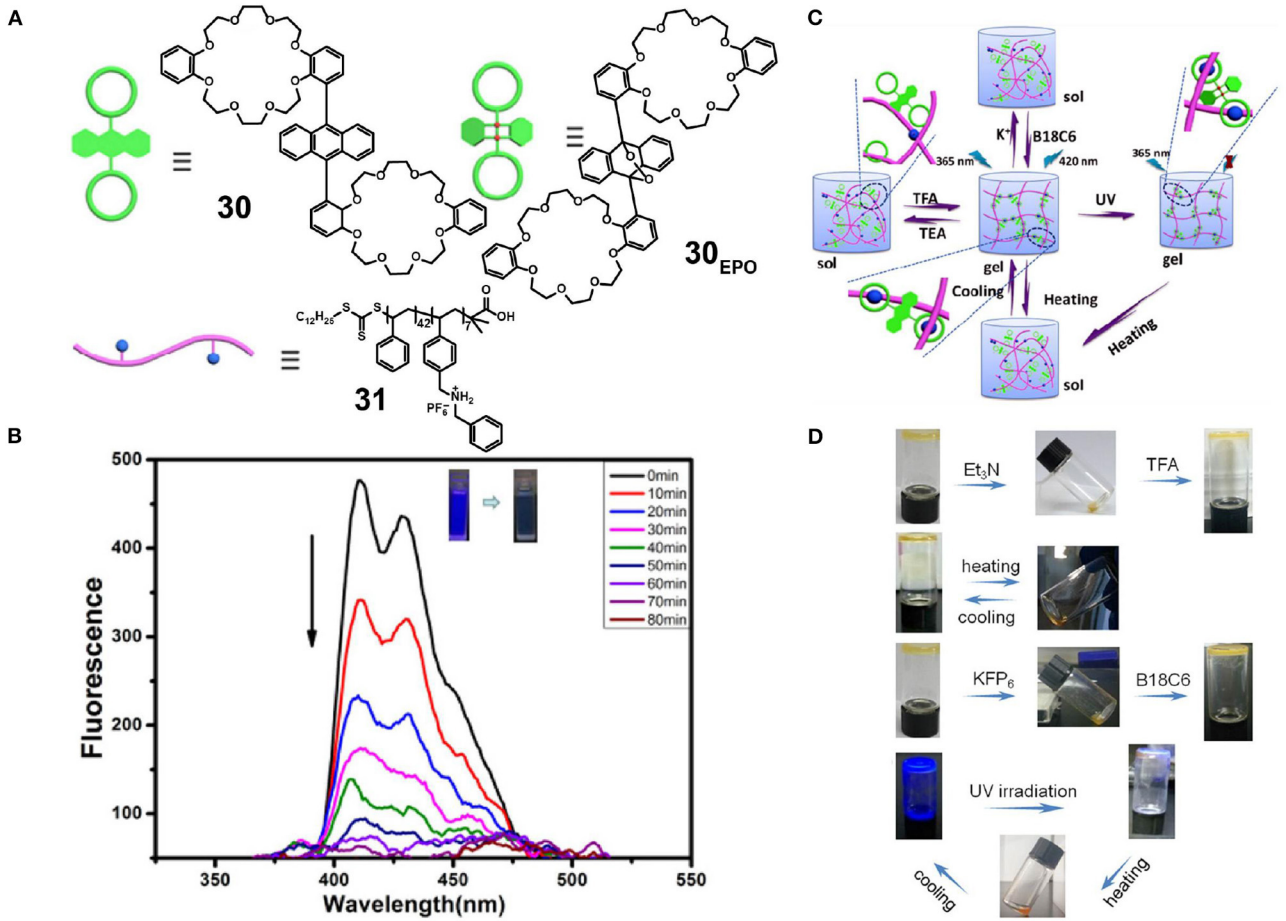
**(A)** Chemical structures and cartoon representations of **30**, **30**_**EPO**_, and **31**. **(B)** Fluorescence emission spectra of **30** irradiated at 365 nm under an O_2_ atmosphere. **(C)** Optical photographs of the reversible gel-sol transition and the reversible light-controlled fluorescence emission behavior. **(D)** Cartoon representations of the reversible gel-sol transition and the reversible light-controlled fluorescence emission behavior. Adapted with permission from Fu et al. (2019); copyright 2019, American Chemical Society.

Fu et al. (2019) also prepared polystyrene **31** containing DBAS units as pendant groups. In addition, they synthesized an anthracene-bridged divalent crown ether **30** (Figure 9A) in which anthracene adopted the *cis*-conformation to prevent π-π stacking between the anthracene units. Upon the simple mixing of **30** and **31** at an appropriately high concentration, a fluorescent supramolecular polymer gel formed based on DB24C8/DBAS recognition, and this polymer displayed a reversible gel-sol transition upon the addition of competitive guest or through pH or thermal stimuli, due to the nature of the dynamic and reversible non-covalent bond (Figures 9C,D). Furthermore, this luminescent supramolecular polymer gel possessed an attractive photo-controlled property due to the fact that host **30**, which contains two bulky groups in the 9 and 10 positions of the anthracene skeleton, could react with singlet oxygen through a [4 + 2] cycloaddition reaction upon irradiation with UV light, therefore leading to a decrease in fluorescence (Figure 9B). This supramolecular system exhibits potential for application in fluorescent materials and, in particular, photo-controlled sensors.

### Polymers as Both Hosts and Guests

**Figure 10:**
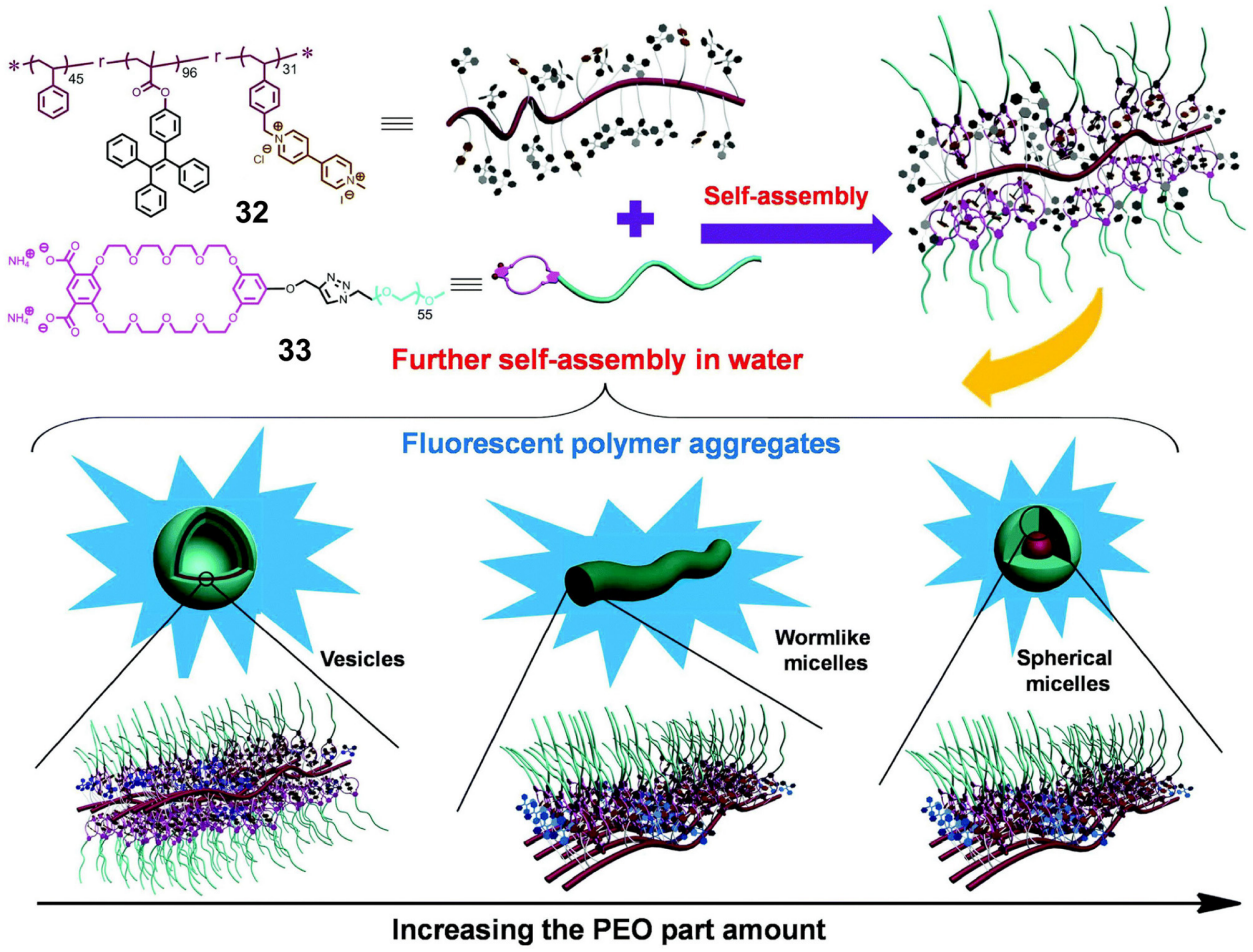
Chemical structures and cartoon representations of **32** and **33**, in addition to cartoon representations of supramolecular graft copolymer construction and further self-assembly into different morphologies. Adapted with permission from Ji X. et al. (2015); copyright 2015, Royal Society of Chemistry.

In the case where polymers are employed both as the hosts and the guests, crown ethers and secondary ammonium salts are both employed to modify traditional polymers. In this context, Ji X. et al. (2015) investigated the influence of the aggregation morphology on the functions of fluorescent polymeric aggregation through constructing supramolecular systems based on hydrophobic polymer **32** and hydrophilic polymer **33** in water. More specifically, polymer **32** consisted of polystyrene with pendent TPE moieties and paraquat units, while polymer **33** consisted of a water-soluble poly(ethylene oxide) (PEO) terminated with a bis-(m-phenylene)-32-crown-10 (BMP32C10) unit containing two COO^−^ groups. Unlike traditional intricate methods for the construction of fluorescent polymeric aggregates through the synthesis of a series of polymers, host-guest interactions between the BMP32C10 and paraquat units in **32** and **33** resulted in the formation of supramolecular amphiphilic graft copolymers. By adjusting the proportion of hydrophilic polymer **33**, different aggregation morphologies, such as vesicles, wormlike micelles, and spherical micelles, can be formed (Figure 10). Thus, due to their amphipathy and special morphology, these supramolecular graft copolymers could encapsulate non-fluorescent drugs and subsequently release these drugs with the appropriate pH stimulus due to the stimulus responsiveness of BMP32C10/paraquat recognition. This could be detected by changes in the intensity of fluorescence. Furthermore, due to the low cytotoxicities of **32** and **33** and the resulting polymer aggregates, this supramolecular system shows promise for application in the field of drug delivery.

## Crown Ether-Based Fluorescent Supramolecular Polymers Constructed by SCCs

**Figure 11:**
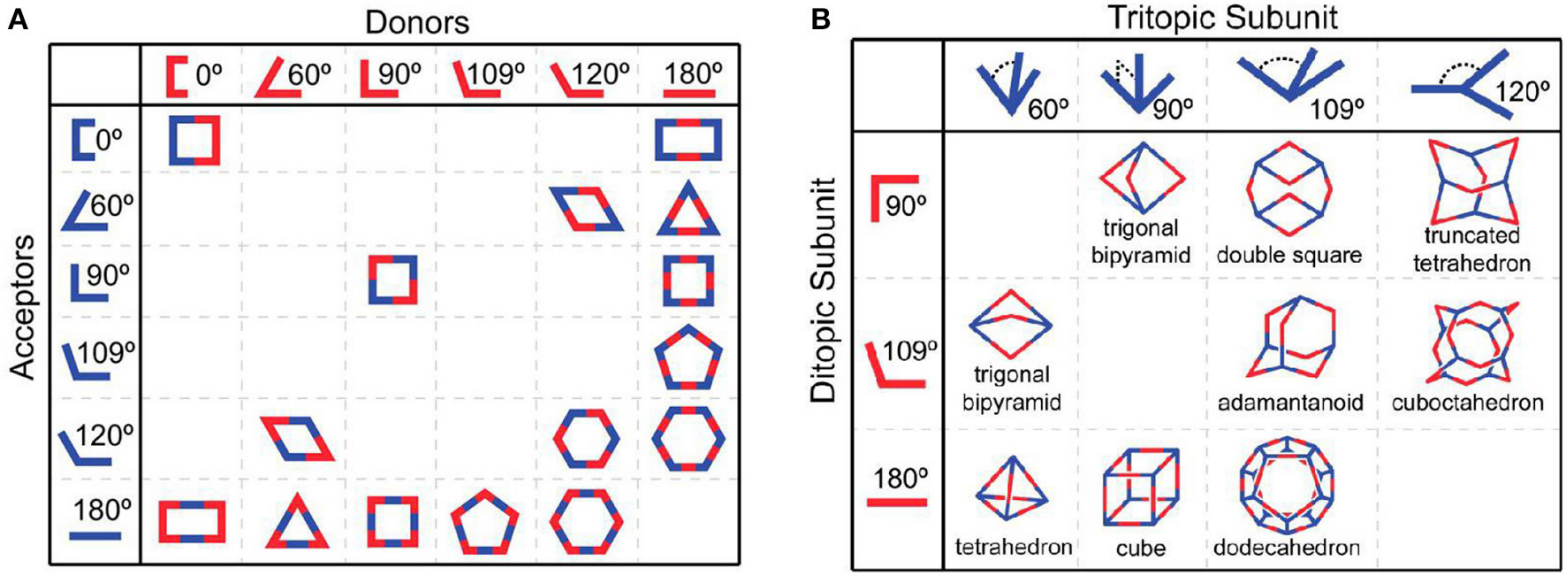
The combination of different building blocks results in **(A)** 2D polygons and **(B)** 3D polygons. Adapted with permission from Cook et al. (2013); copyright 2013, American Chemical Society.

In addition to the aforementioned strategies, discrete supramolecular coordination complexes (SCCs) with well-defined sizes, shapes, and geometries have been proven to be rational building blocks for the construction of fluorescent supramolecular polymers. In a similar manner to natural self-assembly processes, SCCs with superior properties have been efficiently developed by coordination-driven self-assembly, wherein the spontaneous connection between metal acceptors and organic donors via metal-ligand bonds resulted in the formation of non-covalent interactions and complicated structures (Smulders et al., 2013; Wei et al., 2015; Li et al., 2019a; Sun et al., 2019). In addition, over the past few decades, Stang (Sepehrpour et al., 2019), Fujita (Sawada et al., 2014), Raymond (Zhao et al., 2013), Mirkin (Mendez-Arroyo et al., 2014), Newkome (Chakraborty et al., 2017), Nitschke (Mosquera et al., 2016), and Yang (Chen L. -J. et al., 2015) have made enormous contributions to the development of a series of discrete SCCs with various shapes covering two-dimensional metallacycles and three-dimensional metallacages (Figure 11) (Cook et al., 2013). It is necessary to emphasize the difference between the metallic linkage applied in the cases of Figures 1, 2, 8 and that of SCCs. The former could comprise infinite linear polymers or cross-linked networks in which the metal centers and organic ligands bridged through metal-ligand coordination bonds, such as terpyridine-based metal-ligand interactions. The latter could form discrete systems where organometallic receptors and organic donors with specific angularity undergo self-assembly to generate finite supramolecular complexes. Due to the range of supramolecular structures, the stimulus-responsive nature of metal-ligand coordination interactions, and the introduction of metal atoms, SCCs have profound implications for the future development of light-emitting materials (Yan et al., 2015), sensors (Zhang et al., 2017b), molecular flasks (Inokuma et al., 2011), cell imaging (Zhang M. et al., 2016), and bioengineering (Zhou et al., 2019).

In addition, since the formation of metal-ligand coordination interactions does not interfere with other non-covalent interactions, complicated supramolecular polymer systems could be constructed using metal-ligand coordination-based interactions to construct metallacycles or metallacages as repetitive units with other non-covalent interactions, such as crown ether-based recognition systems (Wei et al., 2015). Utilizing two or more non-covalent bond forces to construct supramolecular polymers could not only give them richer stimulus responsiveness and other functions but could also provide a new flexible method for developing the topological structures of supramolecular polymers.

### Metallacycles as Building Blocks

**Figure 12:**
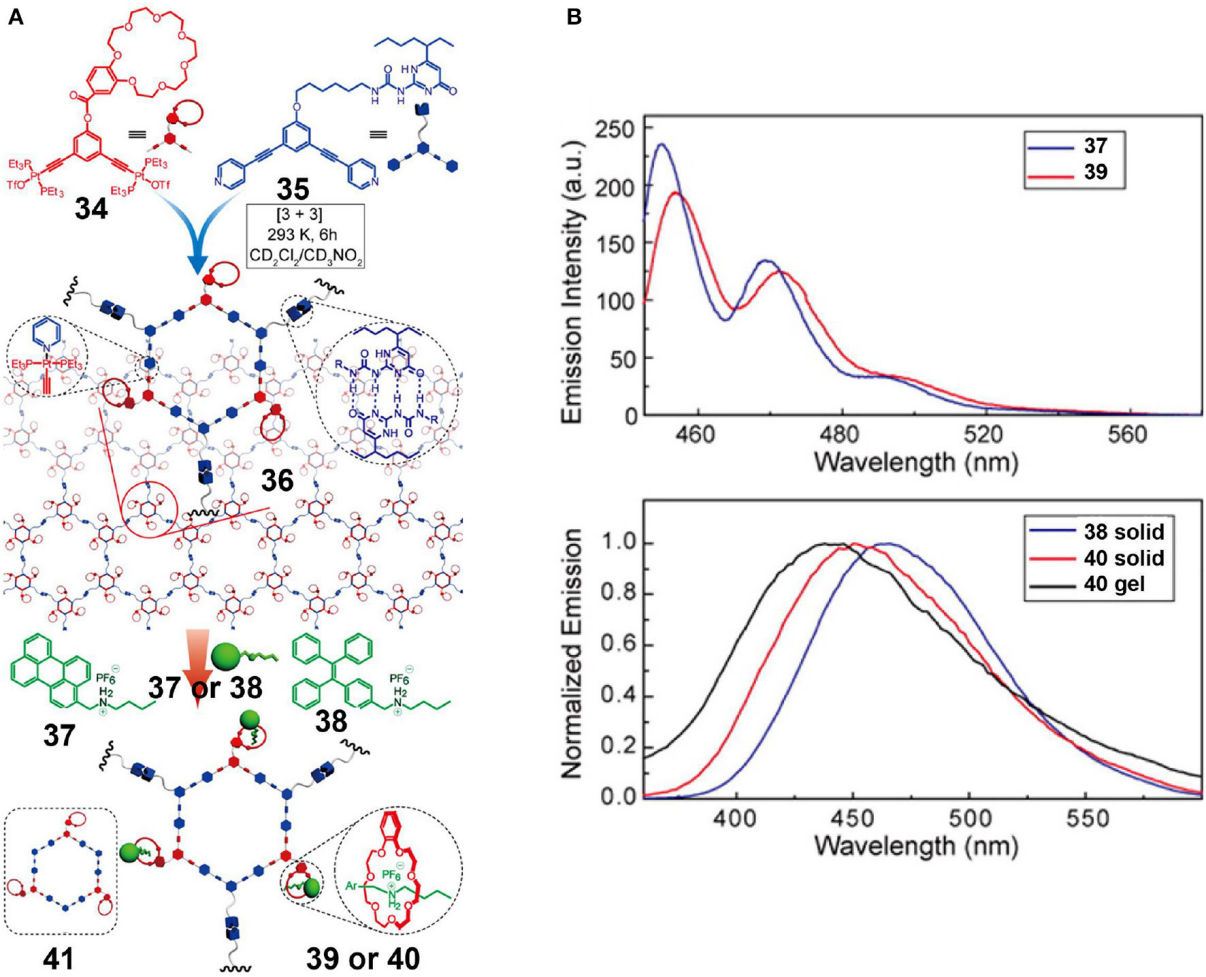
**(A)** Chemical structures of platinum acceptor **34** and dipyridyl donor **35**, and cartoon representation of the construction of the supramolecular polymeric network via triply orthogonal self-assembly. **(B)** Fluorescence emission spectra of **37** and **39** irradiated at 441 nm and fluorescence emission spectra of **38** in the solid state, **40** in the solid state, and **40** in a gel, irradiated at 343 nm. Adapted with permission from Zhou et al. (2016); copyright 2016, American Chemical Society.

Zhou et al. (2016) reported a crown ether-based supramolecular polymer network using three orthogonal interactions, including coordination-driven self-assembly, hydrogen bonding, and host-guest interactions (Figure 12A). More specifically, a platinum acceptor **34** bearing a B21C7 moiety and a corresponding dipyridyl donor **35** with a pendant 2-ureido-4-pyrimidinone (UPy) unit was used to assemble a metallahexagon that was then converted into supramolecular polymer network **36** by the complementary hydrogen-bonding interactions of the UPy units. In addition, the resultant supramolecular polymer network **36** could be functionally modified using the free B21C7 moieties. Driven by host-guest interactions based on the recognition between the B21C7 moieties and dialkylammonium salts, two fluorescent supramolecular polymer networks (**39** and **40**) were developed by the introduction of perylene-decorated (**37**) and TPE-decorated (**38**) dialkylammonium salts, respectively. As evidenced by the fluorescence emission spectra of **37–40** (Figure 12B), the functional supramolecular polymer networks inherited the emission properties of independent precursors in the premise that the preassembly was not disrupted, suggesting that the construction strategy developed here was a feasible, efficient, and imitable methodology for fabricating fluorescent supramolecular polymers through tailored fluorophores.

**Figure 13:**
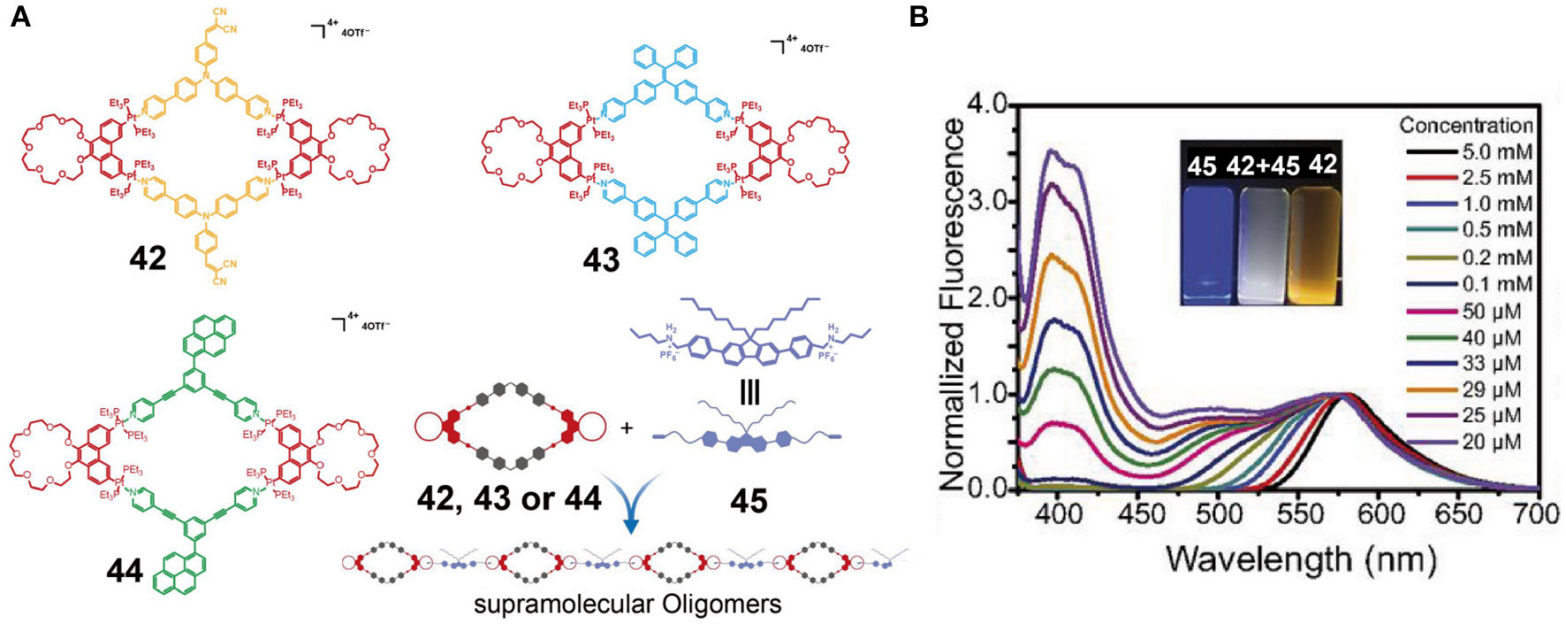
**(A)** Chemical structures of metallacycles **42**, **43**, and **44**, and a cartoon representation of the construction of the supramolecular oligomers. **(B)** Fluorescence emission spectra of equimolar solutions of **42** and **45** at different concentrations. Inset: Optical photographs of solutions of **42** and **45** and of an equimolar mixture of **42** and **45** at a concentration of 29 μM, recorded under UV light (λ_ex_ = 365 nm). Adapted with permission from Zhang et al. (2017b); copyright 2017, National Academy of Sciences (USA).

Zhang et al. (2017b) constructed metallacycles **42–44** by the coordination-driven self-assembly of well-established 120° dipyridyl donors with phenanthrene-21-crown-7 (P21C7)-based 60° diplatinum(II) acceptors. Metallacycles **42–44** exhibited orange, cyan, and green emission colors, respectively, originating from the triphenylamine, tetraphenylethene, and pyrene fluorogens. Further polymerization could be efficiently achieved via crown ether-diaklyammonium salt-based host-guest interactions between the P21C7 units of metallacycles **42–44** and a fluorene-functionalized bis-ammonium salt **45** (Figure 13A). The supramolecular assemblies constructed using **42** and **45** mainly gave blue fluorescence in the dilute solution state (molar concentration of **42** or **45** <25 μM), while they displayed orange emission at high concentrations (molar concentrations of **42** or **45** >0.5 mM) since the ACQ properties of fluorene and the AIE properties of the triphenylamine moiety exhibited strong emissions only in dilute and concentrated solutions, respectively. As a result, this supramolecular system showed a strongly concentration-dependent emission from blue to orange. In addition, at a concentration of 29 μM, this supramolecular system simultaneously displayed white emission because its fluorescence emission covered the entire region from 400 to 700 nm (Figure 13B). Thus, the emission properties of given assemblies can be precise and can also be controlled on a large scale simply by adjusting the system concentration. However, the supramolecular assemblies formed from **43** to **45** and from **44** to **45** did not exhibit a pronounced tuneable emission over a large range due to their similar fluorescence color. Overall, the emissions of such supramolecular systems could be adjusted over a large range by changing the concentration of the system when complementary host-guest interactions (crown-ethers and diammonium salts), complementary emissions (blue and orange), and complementary fluorescence properties (AIE and ACQ) are combined into the same system, thereby providing a simple and effective method for the construction of fluorescent supramolecular assemblies. Such systems therefore play an important role in promoting the application of fluorescent supramolecular assemblies in the field of biotechnology and optoelectronics.

**Figure 14:**
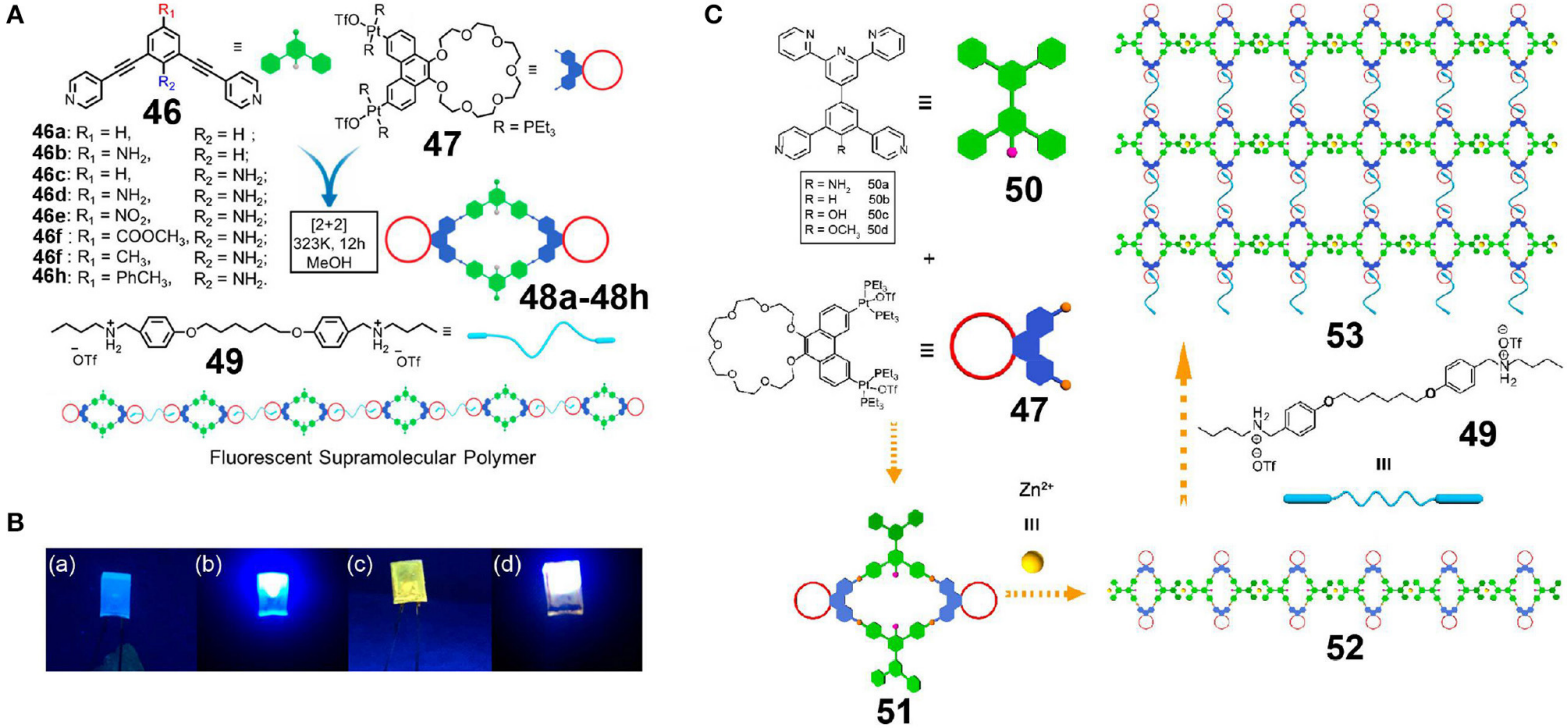
**(A)** Cartoon representations of the construction of supramolecular metallacycles **48a−48h** and the fluorescent supramolecular polymer. **(B)** Optical photographs of the unpainted (a,b) and painted (c,d) UV-LED recorded under UV light (λ_ex_ = 365 nm): (a,c) LED off, (b,d) LED on. Adapted with permission from Xu et al. (2018b); copyright 2018, American Chemical Society. **(C)** Cartoon representation of the construction of supramolecular metallacycles, linear supramolecular polymers, and cross-linked supramolecular polymeric networks. Adapted with permission from Zhang et al. (2019b); copyright 2019, American Chemical Society.

Light-emitting supramolecular assemblies play an important role in the fields of chemical sensors, biological imaging, and organic photoelectric materials. However, control of the fluorescence properties of supramolecular assemblies in a simple way and over a large range is one of the greatest challenges associated with the construction of fluorescent supramolecular assemblies. As an example, Xu et al. (2018b) first synthesized a series of P21C7-functionalized rhomboidal metallacycles **48a−48h** by variation of the substituents present on dipyridyl donor **46** and on P21C7-based 60° diplatinum(II) acceptor **47** (Figure 14A). As a result, the structures of metallacycles **48** differed in the number and position of amino groups on the pyridine ligands or in the electronic effect and the conjugated structure of the aniline moiety at the *para-*position. Metallacycles bearing an endohedral amino group, with strong electron-donating substituents *para* to the aniline group, or presenting a longer conjugated structure, were found to exhibit higher quantum yields both in the solution and thin film states. Fluorescent supramolecular polymers emitting over a wide range from blue to red were then constructed through host-guest interactions between metallacycles **48** and bis-ammonium salts **49**. These supramolecular polymers exhibited higher quantum yields than their corresponding discrete metallacycles both in the solution and thin film states, likely due to the formation of supramolecular polymers inhibiting tight packing of the assemblies, in turn facilitating an alternative stacking pattern that enhanced the emission efficiency. In addition, a white emission light-emitting diode (LED) was obtained by the incorporation of a yellow-emitting supramolecular polymer **48h** with good solubility and a high quantum yield along with a blue-emitting LED source (Figure 14B). The presented system therefore represents a new strategy for the preparation of tuneable fluorescent supramolecular polymers through modification of the functional groups of organic ligands and lays a foundation for the development of self-assembled polymer materials for photoelectric materials, biological imaging, biosensors, and other applications.

The bottom-up method of hierarchical self-assembly based on miscellaneous non-covalent interactions to prepare complex supramolecular structures is a powerful means of building novel functional supramolecular materials. However, the preparation of organometallic materials with precise structural control via hierarchical self-assembly remains a challenge, in particular in the case of systems containing heterometals. For example, Zhang et al. (2019b) reported the construction of four rhomboidal metallacycles **50a−50d** bearing a P21C7 group and a conjugately-linked tripyridine moiety by platinum(II)-ligand coordination-driven self-assembly (Figure 14C). A linear bimetallic supramolecular polymer was then formed through metal-coordination between Zn and the tripyridine moiety. Finally, a bimetallic cross-linked supramolecular polymer was constructed through host-guest interactions between crown-ethers and diammonium salts. These three kinds of orthogonal non-covalent interactions do not interfere with one another, and so at high concentrations, the cross-linked supramolecular polymer can form a gel with multiple-stimulus-responsive properties in addition to good self-healing properties. Furthermore, the emission properties of such supramolecular polymers can be effectively controlled by changing the electron-donor ability of the pyridine ligands present in the metallacycles, thereby providing a new route to novel functional supramolecular polymers through various metal-ligand coordination interactions.

### Metallacages as Building Blocks

**Figure 15:**
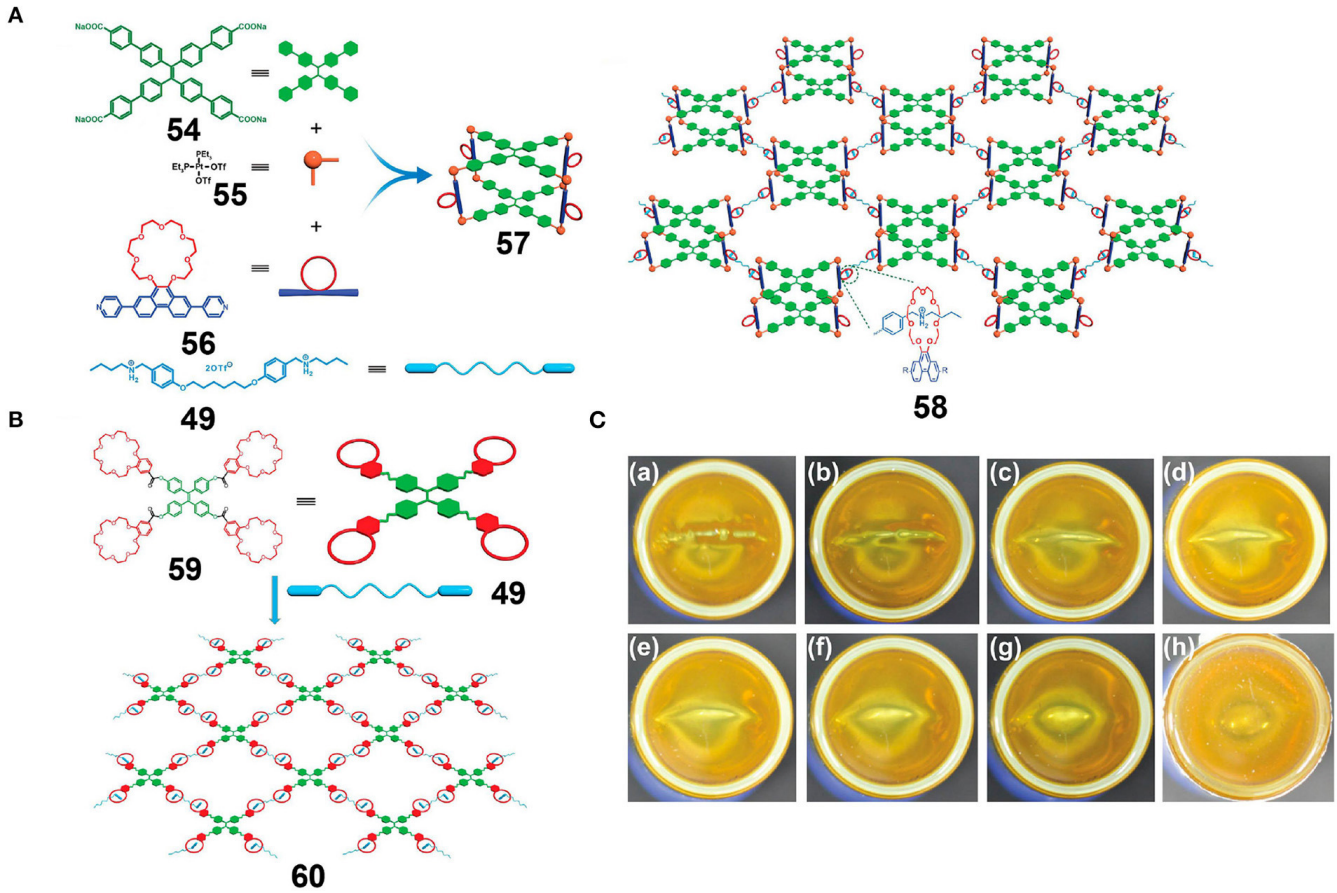
**(A)** Cartoon representations of the construction of supramolecular metallacage **57** and metallacage-cored fluorescent supramolecular polymer **58**. **(B)** Cartoon representation of the construction of cross-linked supramolecular polymer **60**. **(C)** Optical photographs of the self-healing process of gel **58**, and images showing the gel after cutting and allowing to stand for (a) 0, (b) 0.5, (c) 1.0, (d) 1.5, (e) 2, (f) 2.5, (g) 3, and (h) 4 min. Adapted with permission from Lu et al. (2018); copyright 2018, American Chemical Society.

Lu et al. (2018) reported a tetragonal prismatic metallacage, **57**, bearing pendant B21C7 moieties through the incorporation of cis-Pt(PEt_3_)_2_(OTf)_2_, TPE-functionalized sodium benzoate ligands, and linear dipyridyl ligands via metal-coordination-driven self-assembly. Upon the addition of bis-ammonium linkers, a supramolecular polymer network (SPN) was formed via host-guest interactions (Figure 15A), and upon increasing the concentration of the SPN to a relatively high level, a supramolecular polymer gel was obtained that inherited the AIE properties originating from the TPE moieties. Moreover, due to the reversibility and environmental responsiveness of platinum(II)-ligand coordination interactions and the host-guest interactions based on the B21C7 units and bis-ammonium salts, transitions between the metallacage-cored fluorescent supramolecular polymer gel and the disassembled sol with weak fluorescence were achieved by the addition of competing coordination compounds and thermal stimuli. Dynamic and reversible non-covalent interactions endowed the resultant gel with self-healing properties that allowed crack reparation over a short time (Figure 15C). To gain some insight into the effect of metallacages as cores in the gel, a model gel **60** (Figure 15B) was prepared via host-guest interactions between crown ether-functionalized TPE derivatives and bis-ammonium linkers. In comparison with gel **60**, gel **58** displayed a significantly stiffer structure due to the presence of rigid metallacages and higher branch functionalities. Thus, the above system provided a simple yet highly efficient strategy for obtaining multi-functional fluorescent supramolecular gels and laid a foundation for the development of dynamic but robust supramolecular materials. The authors also reported an improvement in the mechanical properties and self-healing properties of these fluorescent supramolecular gel systems when rigid metallacages were introduced through host-guest interactions, thereby showing promise for the application of metal-organic metallacages in intelligent soft materials, luminescent materials, drug carriers, and other fields.

## Conclusions

We herein summarized the current published research works in the field of fluorescent supramolecular polymers formed by crown ether-based host-guest interactions. Depending on the building blocks employed, the fabrication strategies could be divided into three categories, including linking covalently bonded low-molecular weight compounds with the association units of hosts and guests, modifying hosts and guests with polymers, and the use of supramolecular coordination complexes (SCCs) bearing free crown ether units. The examples covered in this review comprehensively describe the superior performances of fluorescent materials based on the marriage of fluorophores and crown ether-based supramolecular macrocyclic compounds, which have demonstrated value in applications such as solid-state or gel-state fluorescent materials, fluorescent sensors, drug delivery monitoring, and optoelectronic devices. Aggregation-induced emission luminogens and conjugated oligomers such as tetraphenylethylene, triphenylamine, and oligomers of fluorene are usually selected to serve as the luminescent groups due to their facile modification and good luminescence. In addition, novel multifunctional supramolecular polymeric materials can be obtained through the combination of crown-ether-based host-guest interactions and other interactions, such as metal-ligand coordination interactions, and multiple hydrogen-bonding interactions. Furthermore, responsiveness to multiple stimuli is a particularly pronounced characteristic of crown ether-based supramolecular polymers, which arises from the dynamic and reversible non-covalent bonding present in such structures. Moreover, crown ether-based fluorescent supramolecular polymers bearing more topological structures possess the desired properties of both rigidity and reversibility, thereby rendering it possible for them to be easily functionalized and developed.

Although there have been important advances in fluorescent supramolecular polymers formed through crown ether-based host-guest interactions, efforts still should be devoted to exploiting uncharted terrains and addressing remaining challenges to ensure more extensive practical applications. Firstly, additional attention should be paid to utilizing existing fluorophores but also to creating novel fluorophores with unique characteristics. Secondly, there is a lack of depth and system in the research into fluorescent supramolecular polymers with various morphologies. However, the construction of amphipathic fluorescent nanoparticles from supramolecular polymers via crown ether-based recognition is one of the developments that could expand the potential applications in biology, such as cell imaging. Moreover, a feasible method for enriching the properties of fluorescent supramolecular polymeric materials is the introduction of more non-covalent interactions, such as π-π stacking interactions, electrostatic interactions, and van der Waals forces. Finally, SCCs and, in particular, metallacages have been proven to be a novel kind of building block that is conducive to the emergence of new fluorescent supramolecular polymers. It can be anticipated that the combination of various fluorescent properties and the unique responsiveness and reversibility of crown ether-based supramolecular macrocyclic chemistry may broaden the application range of developing smart materials in areas such as photocatalysis, information transition, and data storage. We therefore expect continual endeavors to be carried out to ensure the development of novel fluorescent supramolecular polymers that exhibit desirable and enhanced properties as well as to ensure their subsequent practical application.

## Author Contributions

HQ, TH, YL, and SY designed the proposal and revised the manuscript. JZ wrote the first draft of the manuscript and prepared the figures. All authors read and approved the final manuscript prior to submission.

## Conflict of Interest

The authors declare that the research was conducted in the absence of any commercial or financial relationships that could be construed as a potential conflict of interest.

## References

[B1] Bai W. Wang Z. Tong J. Mei J. Qin A. Sun J. Z.. (2015). A self-assembly induced emission system constructed by the host–guest interaction of AIE-active building blocks. Chem. Commun. 51, 1089–1091. 10.1039/C4CC06510G 25449174

[B2] Chakraborty S. Hong W. Endres K. J. Xie T.-Z. Wojtas L. Moorefield C. N.. (2017). Terpyridine-based, flexible tripods: from a highly symmetric nanosphere to temperature-dependent, irreversible, 3D isomeric macromolecular nanocages. J. Am. Chem. Soc. 139, 3012–3020. 10.1021/jacs.6b11784 28165736

[B3] Chen D. Zhan J. Zhang M. Zhang J. Tao J. Tang D. (2015). A fluorescent supramolecular polymer with aggregation induced emission (AIE) properties formed by crown ether-based host–guest interactions. Polym. Chem. 6, 25–29. 10.1039/C4PY01206B

[B4] Chen L.-J. Ren Y.-Y. Wu N.-W. Sun B. Ma J.-Q. Zhang L.. (2015). Hierarchical self-assembly of discrete organoplatinum(II) metallacycles with polysaccharide via electrostatic interactions and their application for heparin detection. J. Am. Chem. Soc. 137, 11725–11735. 10.1021/jacs.5b06565 26322626

[B5] Chen P.-Z. Weng Y.-X. Niu L.-Y. Chen Y.-Z. Wu L.-Z. Tung C.-H.. (2016). Light-harvesting systems based on organic nanocrystals to mimic chlorosomes. Angew. Chem. 128, 2809–2813. 10.1002/ange.201510503 26799735

[B6] Cook T. R. Zheng Y.-R. Stang P. J. (2013). Metal–organic frameworks and self-assembled supramolecular coordination complexes: comparing and contrasting the design, synthesis, and functionality of metal–organic materials. Chem. Rev. 113, 734–777. 10.1021/cr3002824 23121121 PMC3764682

[B7] Deng Y. Zhang Q. Feringa B. L. Tian H. Qu D.-H. (2020). Toughening a self-healable supramolecular polymer by ionic cluster-enhanced iron-carboxylate complexes. Angew. Chem. Int. Ed. 59, 5278–5283. 10.1002/anie.201913893 32096593

[B8] Ding Y. Wang P. Tian Y.-K. Tian Y.-J. Wang F. (2013). Formation of stimuli-responsive supramolecular polymeric assemblies via orthogonal metal–ligand and host–guest interactions. Chem. Commun. 49, 5951–5953. 10.1039/c3cc42511h 23715285

[B9] Dong R. Ravinathan S. P. Xue L. Li N. Zhang Y. Zhou L.. (2016). Dual-responsive aggregation-induced emission-active supramolecular nanoparticles for gene delivery and bioimaging. Chem. Commun. 52, 7950–7953. 10.1039/C6CC02794F 27251637

[B10] Dong S. Zheng B. Xu D. Yan X. Zhang M. Huang F. (2012). A crown ether appended super gelator with multiple stimulus responsiveness. Adv. Mater. 24, 3191–3195. 10.1002/adma.201200837 22605411

[B11] Dsouza R. N. Pischel U. Nau W. M. (2011). Fluorescent dyes and their supramolecular host/guest complexes with macrocycles in aqueous solution. Chem. Rev. 111, 7941–7980. 10.1021/cr200213s 21981343

[B12] Fermi A. Bergamini G. Roy M. Gingras M. Ceroni P. (2014). Turn-on phosphorescence by metal coordination to a multivalent terpyridine ligand: a new paradigm for luminescent sensors. J. Am. Chem. Soc. 136, 6395–6400. 10.1021/ja501458s 24725096

[B13] Fu H.-G. Chen Y. Liu Y. (2019). Multistimuli-responsive and photocontrolled supramolecular luminescent gels constructed by anthracene-bridged bis(dibenzo-24-crown-8) with secondary ammonium salt polymer. ACS Appl. Mater. Interfaces 11, 16117–16122. 10.1021/acsami.9b04323 30983331

[B14] Gibson H. W. Yamaguchi N. Hamilton L. Jones J. W. (2002). Cooperative self-assembly of dendrimers via pseudorotaxane formation from a homotritopic guest molecule and complementary monotopic host dendrons. J. Am. Chem. Soc. 124, 4653–4665. 10.1021/ja012155s 11971714

[B15] Gibson H. W. Yamaguchi N. Jones J. W. (2003). Supramolecular pseudorotaxane polymers from complementary pairs of homoditopic molecules. J. Am. Chem. Soc. 125, 3522–3533. 10.1021/ja020900a 12643714

[B16] He L. Li L. Liu X. Wang J. Huang H. Bu W. (2016). Acid–base-controlled and dibenzylammonium-assisted aggregation induced emission enhancement of poly(tetraphenylethene) with an impressive blue shift. Polym. Chem. 7, 3722–3730. 10.1039/C6PY00275G

[B17] He L. Liang J. Cong Y. Chen X. Bu W. (2014). Concentration and acid–base controllable fluorescence of a metallosupramolecular polymer. Chem. Commun. 50, 10841–10844. 10.1039/C4CC04243C 25089600

[B18] Huang D. Zhang Q. Deng Y. Luo Z. Li B. Shen X. (2018). Polymeric crown ethers: LCST behavior in water and stimuli-responsiveness. Polym. Chem. 9, 2574–2579. 10.1039/C8PY00412A

[B19] Huang F. Gibson H. W. (2004). Formation of a supramolecular hyperbranched polymer from self-organization of an AB2 monomer containing a crown ether and two paraquat Moieties. J. Am. Chem. Soc. 126, 14738–14739. 10.1021/ja044830e 15535696

[B20] Huang F. Nagvekar D. S. Zhou X. Gibson H. W. (2007). Formation of a linear supramolecular polymer by self-assembly of two homoditopic monomers based on the bis(m-phenylene)-32-crown-10/paraquat recognition motif. Macromolecules 40, 3561–3567. 10.1021/ma062080i

[B21] Inokuma Y. Kawano M. Fujita M. (2011). Crystalline molecular flasks. Nat. Chem. 3, 349–358. 10.1038/nchem.1031 21505492

[B22] Ji X. Dong S. Wei P. Xia D. Huang F. (2013a). A novel diblock copolymer with a supramolecular polymer block and a traditional polymer block: preparation, controllable self-assembly in water, and application in controlled release. Adv. Mater. 25, 5725–5729. 10.1002/adma.201301654 23925973

[B23] Ji X. Li Y. Wang H. Zhao R. Tang G. Huang F. (2015). Facile construction of fluorescent polymeric aggregates with various morphologies by self-assembly of supramolecular amphiphilic graft copolymers. Polym. Chem. 6, 5021–5025. 10.1039/C5PY00801H

[B24] Ji X. Yao Y. Li J. Yan X. Huang F. (2013b). A supramolecular cross-linked conjugated polymer network for multiple fluorescent sensing. J. Am. Chem. Soc. 135, 74–77. 10.1021/ja3108559 23259828

[B25] Ji X. F. Wang P. Wang H. Huang F.-H. (2015). A fluorescent supramolecular crosslinked polymer gel formed by crown ether based host-guest interactions and aggregation induced emission. Chin. J. Polym. Sci. 33, 890–898. 10.1007/s10118-015-1639-6

[B26] Laurent J. Blin G. Chatelain F. Vanneaux V. Fuchs A. Larghero J.. (2017). Convergence of microengineering and cellular self-organization towards functional tissue manufacturing. Nat. Biomed. Eng. 1, 939–956. 10.1038/s41551-017-0166-x 31015708

[B27] Li B. He T. Fan Y. Yuan X. Qiu H. Yin S. (2019a). Recent developments in the construction of metallacycle/metallacage-cored supramolecular polymers via hierarchical self-assembly. Chem. Commun. 55, 8036–8059. 10.1039/C9CC02472G 31206102

[B28] Li B. He T. Shen X. Tang D. Yin S. (2019b). Fluorescent supramolecular polymers with aggregation induced emission properties. Polym. Chem. 10, 796–818. 10.1039/C8PY01396A

[B29] Li B. Lin C. Lu C. Zhang J. He T. Qiu H. (2020). A rapid and reversible thermochromic supramolecular polymer hydrogel and its application in protected quick response codes. Mater. Chem. Front. 4, 869–874. 10.1039/C9QM00699K

[B30] Li J. Shi K. Drechsler M. Tang B. Z. Huang J. Yan Y. (2016). A supramolecular fluorescent vesicle based on a coordinating aggregation induced emission amphiphile: insight into the role of electrical charge in cancer cell division. Chem. Commun. 52, 12466–12469. 10.1039/C6CC06432A 27711439

[B31] Li X. Wang L. Deng Y. Luo Z. Zhang Q. Dong S.. (2018). Preparation of cross-linked supramolecular polymers based on benzo-21-crown-7/secondary ammonium salt host–guest interactions. Chem. Commun. 54, 12459–12462. 10.1039/C8CC07657J 30335096

[B32] Li Y. Dong Y. Miao X. Ren Y. Zhang B. Wang P. (2018). Shape-controllable and fluorescent supramolecular organic frameworks through aqueous host–guest complexation. Angew. Chem. Int. Ed. 57, 729–733. 10.1002/anie.201710553 29193530

[B33] Liu Y. Zhang Y. Zhu H. Wang H. Tian W. Shi B. (2018). A supramolecular hyperbranched polymer with multi-responsiveness constructed by pillar[5]arene-based host–guest recognition and its application in the breath figure method. Mater. Chem. Front. 2, 1568–1573. 10.1039/C8QM00220G

[B34] Lou X.-Y. Yang Y.-W. (2018). Manipulating aggregation-induced emission with supramolecular macrocycles. Adv. Optical Mater. 6:1800668 10.1002/adom.201800668

[B35] Lu C. Zhang M. Tang D. Yan X. Zhang Z. Zhou Z.. (2018). Fluorescent metallacage-core supramolecular polymer gel formed by orthogonal metal coordination and host–guest interactions. J. Am. Chem. Soc. 140, 7674–7680. 10.1021/jacs.8b03781 29856215 PMC6385593

[B36] Luo J. Xie Z. Lam J. W. Y. Cheng L. Chen H. Qiu C.. (2001). Aggregation-induced emission of 1-methyl-1,2,3,4,5-pentaphenylsilole. Chem. Commun., 1740–1741. 10.1039/b105159h 12240292

[B37] Ma X. Xie J. Tang N. Wu J. (2016). AIE-caused luminescence of a thermally-responsive supramolecular organogel. New J. Chem. 40, 6584–6587. 10.1039/C6NJ01211F

[B38] Ma X. Zhao Y. (2015). Biomedical applications of supramolecular systems based on host–guest interactions. Chem. Rev. 115, 7794–7839. 10.1021/cr500392w 25415447

[B39] Ma Y. Marszalek T. Yuan Z. Stangenberg R. Pisula W. Chen L.. (2015). A crown ether decorated dibenzocoronene tetracarboxdiimide chromophore: synthesis, sensing, and self-organization. Chem. Asian J. 10, 139–143. 10.1002/asia.201403037 25319139

[B40] Mendez-Arroyo J. Barroso-Flores J. Lifschitz A. M. Sarjeant A. A. Stern C. L. Mirkin C. A. (2014). A multi-state, allosterically-regulated molecular receptor with switchable selectivity. J. Am. Chem. Soc. 136, 10340–10348. 10.1021/ja503506a 25007350

[B41] Mosquera J. Ronson T. K. Nitschke J. R. (2016). Subcomponent flexibility enables conversion between D4-symmetric ${\text{Cd}}_{8}^{\text{II}}$L_8_ and T-symmetric ${\text{Cd}}_{4}^{\text{II}}$L_4_ assemblies. J. Am. Chem. Soc. 138, 1812–1815. 10.1021/jacs.5b12955 26814599

[B42] Pedersen C. J. (1967). Cyclic polyethers and their complexes with metal salts. J. Am. Chem. Soc. 89, 7017–7036. 10.1021/ja01002a035

[B43] Peng H.-Q. Zheng X. Han T. Kwok R. T. K. Lam J. W. Y. Huang X.. (2017). Dramatic differences in aggregation-induced emission and supramolecular polymerizability of tetraphenylethene-based stereoisomers. J. Am. Chem. Soc. 139, 10150–10156. 10.1021/jacs.7b05792 28692263

[B44] Qu D.-H. Wang Q.-C. Zhang Q.-W. Ma X. Tian H. (2015). Photoresponsive host–guest functional systems. Chem. Rev. 115, 7543–7588. 10.1021/cr5006342 25697681

[B45] Roy B. Noguchi T. Yoshihara D. Yamamoto T. Sakamoto J. Shinkai S. (2016). Amplified fluorescence emission of bolaamphiphilic perylene-azacrown ether derivatives directed towards molecular recognition events. PCCP 18, 13239–13245. 10.1039/C6CP01545J 27118684

[B46] Sawada T. Hisada H. Fujita M. (2014). Mutual induced fit in a synthetic host–guest system. J. Am. Chem. Soc. 136, 4449–4451. 10.1021/ja500376x 24611612

[B47] Sepehrpour H. Fu W. Sun Y. Stang P. J. (2019). Biomedically relevant self-assembled metallacycles and metallacages. J. Am. Chem. Soc. 141, 14005–14020. 10.1021/jacs.9b06222 31419112 PMC6744948

[B48] Shi B. Jie K. Zhou Y. Zhou J. Xia D. Huang F. (2016). Nanoparticles with near-infrared emission enhanced by pillararene-based molecular recognition in water. J. Am. Chem. Soc. 138, 80–83. 10.1021/jacs.5b11676 26699758

[B49] Shi B. Zhou Z. Vanderlinden R. T. Tang J.-H. Yu G. Acharyya K.. (2019). Spontaneous supramolecular polymerization driven by discrete platinum metallacycle-based host–guest complexation. J. Am. Chem. Soc. 141, 11837–11841. 10.1021/jacs.9b06181 31303001 PMC6693626

[B50] Shi C.-Y. Zhang Q. Yu C.-Y. Rao S.-J. Yang S. Tian H.. (2020). An ultrastrong and highly stretchable polyurethane elastomer enabled by a zipper-like ring-sliding effect. Adv. Mater. 32:2000345. 10.1002/adma.202000345 32350950

[B51] Smulders M. M. J. Riddell I. A. Browne C. Nitschke J. R. (2013). Building on architectural principles for three-dimensional metallosupramolecular construction. Chem. Soc. Rev. 42, 1728–1754. 10.1039/C2CS35254K 23032789

[B52] Sun Y. Chen C. Stang P. J. (2019). Soft materials with diverse suprastructures via the self-assembly of metal–organic complexes. Acc. Chem. Res. 52, 802–817. 10.1021/acs.accounts.8b00663 30794371 PMC6497072

[B53] Wang H. Ji X. Li Z. Zhu C. N. Yang X. Li T. (2017). Preparation of a white-light-emitting fluorescent supramolecular polymer gel with a single chromophore and use of the gel to fabricate a protected quick response code. Mater. Chem. Front. 1, 167–171. 10.1039/C6QM00164E

[B54] Wang L. Cheng L. Li G. Liu K. Zhang Z. Li P.. (2020). A self-cross-linking supramolecular polymer network enabled by crown-ether-based molecular recognition. J. Am. Chem. Soc. 142, 2051–2058. 10.1021/jacs.9b12164 31905287

[B55] Wang X. Hu J. Liu T. Zhang G. Liu S. (2012). Highly sensitive and selective fluorometric off–on K+ probe constructed via host–guest molecular recognition and aggregation-induced emission. J. Mater. Chem. 22, 8622–8628. 10.1039/c2jm16510d

[B56] Wei P. Yan X. Huang F. (2015). Supramolecular polymers constructed by orthogonal self-assembly based on host–guest and metal–ligand interactions. Chem. Soc. Rev. 44, 815–832. 10.1039/C4CS00327F 25423355

[B57] Xiao T. Zhou L. Sun X.-Q. Huang F. Lin C. Wang L. (2020). Supramolecular polymers fabricated by orthogonal self-assembly based on multiple hydrogen bonding and macrocyclic host–guest interactions. Chin. Chem. Lett. 31, 1–9. 10.1016/j.cclet.2019.05.011

[B58] Xu L. Chen D. Zhang Q. He T. Lu C. Shen X. (2018a). A fluorescent cross-linked supramolecular network formed by orthogonal metal-coordination and host–guest interactions for multiple ratiometric sensing. Polym. Chem. 9, 399–403. 10.1039/C7PY01788J

[B59] Xu L. Shen X. Zhou Z. He T. Zhang J. Qiu H.. (2018b). Metallacycle-cored supramolecular polymers: fluorescence tuning by variation of substituents. J. Am. Chem. Soc. 140, 16920–16924. 10.1021/jacs.8b10842 30465423 PMC6469999

[B60] Yamaguchi N. Gibson H. W. (1999). Formation of supramolecular polymers from homoditopic molecules containing secondary ammonium ions and crown ether moieties. Angew. Chem. Int. Ed. 38, 143–147.

[B61] Yamaguchi N. Nagvekar D. S. Gibson H. W. (1998). Self-organization of a heteroditopic molecule to linear polymolecular arrays in solution. Angew. Chem. Int. Ed. 37, 2361–2364. 29710954 10.1002/(SICI)1521-3773(19980918)37:17<2361::AID-ANIE2361>3.0.CO;2-P

[B62] Yan X. Cook T. R. Pollock J. B. Wei P. Zhang Y. Yu Y.. (2014). Responsive supramolecular polymer metallogel constructed by orthogonal coordination-driven self-assembly and host/guest interactions. J. Am. Chem. Soc. 136, 4460–4463. 10.1021/ja412249k 24621148

[B63] Yan X. Cook T. R. Wang P. Huang F. Stang P. J. (2015). Highly emissive platinum(II) metallacages. Nat. Chem. 7, 342–348. 10.1038/nchem.2201 25803473

[B64] Yan X. Li S. Cook T. R. Ji X. Yao Y. Pollock J. B.. (2013). Hierarchical self-assembly: well-defined supramolecular nanostructures and metallohydrogels via amphiphilic discrete organoplatinum(II) metallacycles. J. Am. Chem. Soc. 135, 14036–14039. 10.1021/ja406877b 23927740

[B65] Yan X. Wang F. Zheng B. Huang F. (2012a). Stimuli-responsive supramolecular polymeric materials. Chem. Soc. Rev. 41, 6042–6065. 10.1039/c2cs35091b 22618080

[B66] Yan X. Xu D. Chi X. Chen J. Dong S. Ding X.. (2012b). A multiresponsive, shape-persistent, and elastic supramolecular polymer network gel constructed by orthogonal self-assembly. Adv. Mater. 24, 362–369. 10.1002/adma.201103220 22161963

[B67] Yu G. Jie K. Huang F. (2015). Supramolecular amphiphiles based on host–guest molecular recognition motifs. Chem. Rev. 115, 7240–7303. 10.1021/cr5005315 25716119

[B68] Yu G. Yan X. Han C. Huang F. (2013). Characterization of supramolecular gels. Chem. Soc. Rev. 42, 6697–6722. 10.1039/c3cs60080g 23744396

[B69] Yu G. Zhang M. Saha M. L. Mao Z. Chen J. Yao Y.. (2017). Antitumor activity of a unique polymer that incorporates a fluorescent self-assembled metallacycle. J. Am. Chem. Soc. 139, 15940–15949. 10.1021/jacs.7b09224 29019660 PMC5827967

[B70] Yu X. Chen L. Zhang M. Yi T. (2014). Low-molecular-mass gels responding to ultrasound and mechanical stress: towards self-healing materials. Chem. Soc. Rev. 43, 5346–5371. 10.1039/C4CS00066H 24770929

[B71] Zhan J. Li Q. Hu Q. Wu Q. Li C. Qiu H.. (2014a). A stimuli-responsive orthogonal supramolecular polymer network formed by metal–ligand and host–guest interactions. Chem. Commun. 50, 722–724. 10.1039/C3CC47468B 24287616

[B72] Zhan J. Zhang M. Zhou M. Liu B. Chen D. Liu Y.. (2014b). A multiple-responsive self-healing supramolecular polymer gel network based on multiple orthogonal interactions. Macromol. Rapid Commun. 35, 1424–1429. 10.1002/marc.201400216 24943122

[B73] Zhang C. Li S. Zhang J. Zhu K. Li N. Huang F. (2007). Benzo-21-crown-7/secondary dialkylammonium salt [2]pseudorotaxane- and [2]rotaxane-type threaded structures. Org. Lett. 9, 5553–5556. 10.1021/ol702510c 18047364

[B74] Zhang J. Zhang K. Huang X. Cai W. Zhou C. Liu S. (2012). Supramolecular light-emitting polymers for solution-processed optoelectronic devices. J. Mater. Chem. 22, 12759–12766. 10.1039/c2jm31773g

[B75] Zhang J. Zhu J. Lu C. Gu Z. He T. Yang A. (2016). A hyperbranched fluorescent supramolecular polymer with aggregation induced emission (AIE) properties. Polym. Chem. 7, 4317–4321. 10.1039/C6PY00872K

[B76] Zhang M. Li S. Yan X. Zhou Z. Saha M. L. Wang Y.-C.. (2016). Fluorescent metallacycle-cored polymers via covalent linkage and their use as contrast agents for cell imaging. Proc. Natl. Acad. Sci. U.S.A. 113:11100. 10.1073/pnas.1612898113 27647900 PMC5056100

[B77] Zhang M. Saha M. L. Wang M. Zhou Z. Song B. Lu C.. (2017a). Multicomponent platinum(II) cages with tunable emission and amino acid sensing. J. Am. Chem. Soc. 139, 5067–5074. 10.1021/jacs.6b12536 28332834

[B78] Zhang M. Yin S. Zhang J. Zhou Z. Saha M. L. Lu C.. (2017b). Metallacycle-cored supramolecular assemblies with tunable fluorescence including white-light emission. Proc. Natl. Acad. Sci. U.S.A. 114:3044. 10.1073/pnas.1702510114 28265080 PMC5373375

[B79] Zhang Q. Deng Y.-X. Luo H.-X. Shi C.-Y. Geise G. M. Feringa B. L.. (2019a). Assembling a natural small molecule into a supramolecular network with high structural order and dynamic functions. J. Am. Chem. Soc. 141, 12804–12814. 10.1021/jacs.9b05740 31348651 PMC6696886

[B80] Zhang Q. Shi C.-Y. Qu D.-H. Long Y.-T. Feringa B. L. Tian H. (2018). Exploring a naturally tailored small molecule for stretchable, self-healing, and adhesive supramolecular polymers. Sci. Adv. 4:eaat8192. 10.1126/sciadv.aat8192 30062126 PMC6063538

[B81] Zhang Q. Tang D. Zhang J. Ni R. Xu L. He T.. (2019b). Self-Healing heterometallic supramolecular polymers constructed by hierarchical assembly of triply orthogonal interactions with tunable photophysical properties. J. Am. Chem. Soc. 141, 17909–17917. 10.1021/jacs.9b09671 31617714 PMC7001772

[B82] Zhao C. Sun Q.-F. Hart-Cooper W. M. DiPasquale A. G. Toste F. D. Bergman R. G.. (2013). Chiral amide directed assembly of a diastereo- and enantiopure supramolecular host and its application to enantioselective catalysis of neutral substrates. J. Am. Chem. Soc. 135, 18802–18805. 10.1021/ja411631v 24283463

[B83] Zheng B. Wang F. Dong S. Huang F. (2012). Supramolecular polymers constructed by crown ether-based molecular recognition. Chem. Soc. Rev. 41, 1621–1636. 10.1039/C1CS15220C 22012256

[B84] Zhou Z. Liu J. Huang J. Rees T. W. Wang Y. Wang H.. (2019). A self-assembled Ru–Pt metallacage as a lysosome-targeting photosensitizer for 2-photon photodynamic therapy. Proc. Natl. Acad. Sci. U.S.A. 116:20296. 10.1073/pnas.1912549116 31548389 PMC6789806

[B85] Zhou Z. Yan X. Cook T. R. Saha M. L. Stang P. J. (2016). Engineering functionalization in a supramolecular polymer: hierarchical self-organization of triply orthogonal non-covalent interactions on a supramolecular coordination complex platform. J. Am. Chem. Soc. 138, 806–809. 10.1021/jacs.5b12986 26761393

